# An HIV-1 broadly neutralizing antibody overcomes structural and dynamic variation through highly focused epitope targeting

**DOI:** 10.1038/s44298-023-00002-4

**Published:** 2023-10-05

**Authors:** Edgar A. Hodge, Ananya Chatterjee, Chengbo Chen, Gajendra S. Naika, Mint Laohajaratsang, Vidya Mangala Prasad, Kelly K. Lee

**Affiliations:** 1 Department of Medicinal Chemistry, University of Washington, Seattle, WA 98195 USA; 2 Molecular Biophysics Unit, Indian Institute of Science, Bangalore, Karnataka 560012 India; 3 Biological Physics, Structure and Design Graduate Program, University of Washington, Seattle, WA 98195 USA; 4 Center for Infectious Diseases Research, Indian Institute of Science, Bangalore, Karnataka 560012 India

**Keywords:** Biochemistry, Immunology, Microbiology

## Abstract

The existence of broadly cross-reactive antibodies that can neutralize diverse HIV-1 isolates (bnAbs) has been appreciated for more than a decade. Many high-resolution structures of bnAbs, typically with one or two well-characterized HIV-1 Env glycoprotein trimers, have been reported. However, an understanding of how such antibodies grapple with variability in their antigenic targets across diverse viral isolates has remained elusive. To achieve such an understanding requires first characterizing the extent of structural and antigenic variation embodied in Env, and then identifying how a bnAb overcomes that variation at a structural level. Here, using hydrogen/deuterium-exchange mass spectrometry (HDX-MS) and quantitative measurements of antibody binding kinetics, we show that variation in structural ordering in the V1/V2 apex of Env across a globally representative panel of HIV-1 isolates has a marked effect on antibody association rates and affinities. We also report cryo-EM reconstructions of the apex-targeting PGT145 bnAb bound to two divergent Env that exhibit different degrees of structural dynamics throughout the trimer structures. Parallel HDX-MS experiments demonstrate that PGT145 bnAb has an exquisitely focused footprint at the trimer apex where binding did not yield allosteric changes throughout the rest of the structure. These results demonstrate that structural dynamics are a cryptic determinant of antigenicity, and mature antibodies that have achieved breadth and potency in some cases are able to achieve their broad cross-reactivity by “threading the needle” and binding in a highly focused fashion, thus evading and overcoming the variable properties found in Env from divergent isolates.

## Introduction

The HIV-1 envelope glycoprotein (Env) mediates essential processes of receptor binding and membrane fusion to initiate infection of a host cell. It is the sole target for neutralizing antibodies and thus is under intense immune selection pressures. As a result, Env is the most variable, rapidly evolving part of HIV-1. Env sequences can vary by more than 30% while maintaining conserved receptor usage and cell entry functionality^1^. While we have long been aware of the astounding sequence variation in the Env gene, the structural and functional implications of this diversity are only beginning to be examined and grasped. Structural variation in Env impacts its interactions with all key drivers of viral fitness and replication, and this is not captured solely by variation in sequence. These differences underlie viral phenotypic traits such as neutralization sensitivity, tropism, infectivity, and transmissibility. While recent studies have reported high-resolution structures of the Env trimer, they represent static, platonic ideals of the Env assembly. Under native conditions, HIV-1 Env is a dynamic fusion protein complex that can flicker between antigenically and functionally distinct conformational states, even in the absence of receptor engagement^2–6^. This propensity to undergo large-scale dynamic movements has been shown to be highly isolate-specific in nature. Probing the impact of these intrinsic dynamic traits on Env-antibody recognition is essential for understanding the underlying basis for differences in antigenic and neutralization profiles among HIV-1 isolates. We posit that in order to understand how the most desirable antibodies achieve broad cross-reactivity, it is necessary to both identify the conserved features that are recognized as well as to understand how the antibody copes with the variable contexts in which the conserved features are embedded and presented.

We recently reported the use of a structural mass spectrometry approach, Hydrogen/Deuterium-exchange Mass Spectrometry (HDX-MS) to investigate local structural and dynamic differences in a set of native-like HIV-1 Env trimers^2,7^. The Env trimers in that set like most that have been studied, exemplified by BG505^8^, were selected for study due to their robust production of native-like trimers expressed in cell culture and favorable antigenic properties^8–12^. Despite those common traits, our analysis revealed that many of the regions on Env showed significant differences in local structural ordering and stability that map to conformational epitopes that are targeted by bnAbs^2^.

To assess antigenic variation across isolates more representative of HIV-1 diversity, here we examined native-like Env trimers based upon isolates that comprise a large panel of neuralization resistant isolates that largely capture HIV-1’s antigenic diversity^1,13^. Additional trimers from well-characterized reference isolates such as BG505, JR-FL, B41, and AMC008 were also included in the comparative analysis^8,9,11,14^. Using HDX-MS, we document a dramatic degree of variation in local epitope dynamics in the V1/V2 trimer apex across this expanded panel of diverse isolates. Bio-layer interferometry was used to measure binding kinetics for an apex-targeting bnAb, PGT145, revealing a clear, inverse correlation of association rates and local epitope dynamics.

From structures of antibody Fabs bound to the well-characterized clade A BG505 SOSIP trimer, it has been recognized that the epitope for bnAbs such as PGT145, PG9/16, and others bind to a quaternary epitope involving a basic residue charge “sink” as well as the conserved N160 glycan from more than one gp120 subunit^15–17^. Likewise, the glycan at N156 has been implicated in playing an important role in binding of many of these apex antibodies, but in structures this glycan is frequently not resolved, thus it has been inferred to play an indirect role in positioning and stabilizing adjacent features that themselves are directly recognized by the antibodies^18^.

To map the structural impact of PGT145 binding and understand how it copes with structural and dynamic variation among Env, we performed differential HDX-MS and obtained cryo-EM structures of the PGT145 Fab in complex with Env trimers derived from two HIV-1 isolates whose trimers embody distinct structural dynamic profiles. The results underscore the exquisitely focused targeting of the epitope by this bnAb and also revealed a more complete picture of how the conserved N160 and N156 glycans at the Env apex are coordinated by antibody residues that undergo somatic hypermutation resulting in the mature PGT145 bnAb^19^.

These results demonstrate that local epitope dynamics are a cryptic determinant of HIV-1 Env antigenicity and highlight that, for antibodies targeting the Env apex, breadth can be achieved by selectively targeting an extremely localized protein footprint along with conserved glycans, thus avoiding contributions from structural and dynamic variation among diverse Env.

## Results

### Envs derived from viruses across the global panel of neutralization resistant isolates exhibit significant dynamic variation in the V1/V2 apex

**Fig. 1:**
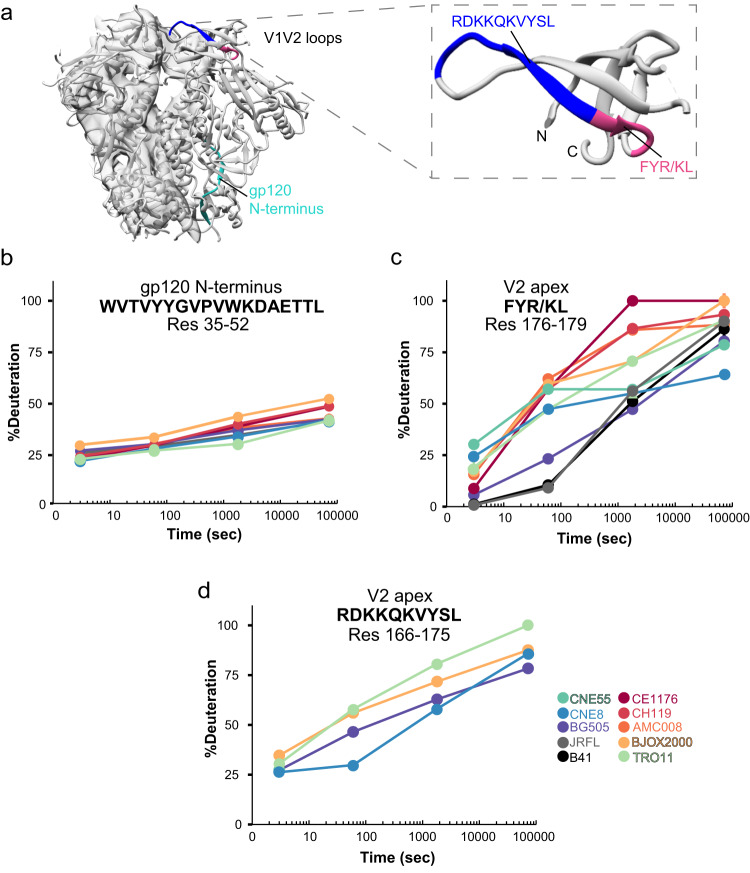
HDX-MS signatures across V2 loop peptides identify differences in V2 bnAb epitope dynamics among diverse native-like trimers from the global panel of tier 2 neutralization resistant isolates and reference strains. **a** V1/V2 loops from the three protomers converge at the trimer apex in prefusion closed Env structures. Two homologous V2 loop peptides that can be tracked by HDX-MS across the panel of Env trimers we examined are highlighted in red and blue in the ribbon diagram. A gp120 N-terminal peptide that is integral to gp120-gp41 association serves as a reporter of trimer integrity, highlighted in teal. **b** Deuterium uptake plot of the gp120 N-terminal peptide shows conserved dynamic behavior among all native-like trimers from all isolates. By contrast, deuterium uptake plots for the two V2 peptides (**c**) segment spanning residues 176–179 and (**d**) spanning residues 166-175 reveal dramatic differences in V2 loop dynamics and local structural ordering across the panel of HIV trimers. Each point in the HDX-MS uptake plots reflect the average percent deuteration of at least two replicates after 3 s, 1 min, 30 min, and 20 h of deuterium exchange normalized to a fully deuterated control. Error bars for standard deviations are shown, however in most cases the magnitude of error is smaller than the size of the symbols shown.

The apex of the HIV-1 Env trimer is composed of V1 and V2 loops layered on top of the V3 loop (Fig. 1a). While certain elements of these so-called “hypervariable loops”, notably at turns in the loops that project from the trimer exhibit high sequence variability, the core structural elements are relatively conserved as they serve important roles in mediating contacts between protomers and packing interactions with the rest of the trimer that help to maintain the prefusion conformation. We used HDX-MS to probe the local structural dynamics of the V1/V2 apex across highly diverse Env which were derived from a global panel of isolates that captures the majority of HIV-1 antigenic variation across all major subtypes and circulating recombinant forms^13^. We examined whether this desirable bnAb epitope exhibits differences in structural ordering and stability that can impact antibody recognition.

By HDX-MS, we observed dramatic differences in dynamics across many homologous peptides, particularly in nearly all regions important for bnAb and receptor binding (manuscript in preparation). Here, we focus on the V1/V2 apex that is a key site of vulnerability targeted by bnAbs. In contrast to peptides such as one located in the N-terminus of gp120 that showed nearly identical deuterium exchange kinetics over the course of the incubation (Fig. 1b), indicating all the trimers were well-formed and intact^4,20^, a key homologous V2 loop peptide spanning residues 176–179 (peptide FYR/KL; Fig. 1c), showed dramatic differences in deuteration kinetics over time across the panel of isolates. Interestingly, at the earliest time point (3 s) deuteration levels were similar, suggesting that the motif in disparate trimers exists in a similar ground state conformation. However, over the course of the incubation, the rates of deuterium uptake diverged dramatically between isolates. This divergence is indicative of the homologous peptide experiencing differences in local ordering and stability, and transiently sampling more exposed conformations to different extents. After 20 h in deuterated buffer, most isolates approach a similar saturated endpoint. In CNE8, however, the peptide remains considerably more protected than the other isolates, indicating that the apex maintains a conformation with less overall exposure of this peptide’s amide backbone. Another key region in V2 is the peptide segment containing residues 166–175 (peptide RDKKQKVYSL; Fig. 1d). Residues in this region are part of the V2 loop C strand containing basic residues important for binding both PGT145 as well as other apex-targeting bnAbs such as CAP256-VRC26^15,17,21,22^. This homologous peptide was found across a smaller number of Envs than the adjacent FYK/RL peptide, however similar dynamic trends were observed, and again CNE8 as well as BG505 exhibited reduced deuterium uptake kinetics compared to other Env including TRO11 and BJOX2000, suggesting substantially greater local structural ordering in some Env trimers.

### The impact of V2 epitope dynamics on bnAb recognition and binding kinetics

**Fig. 2:**
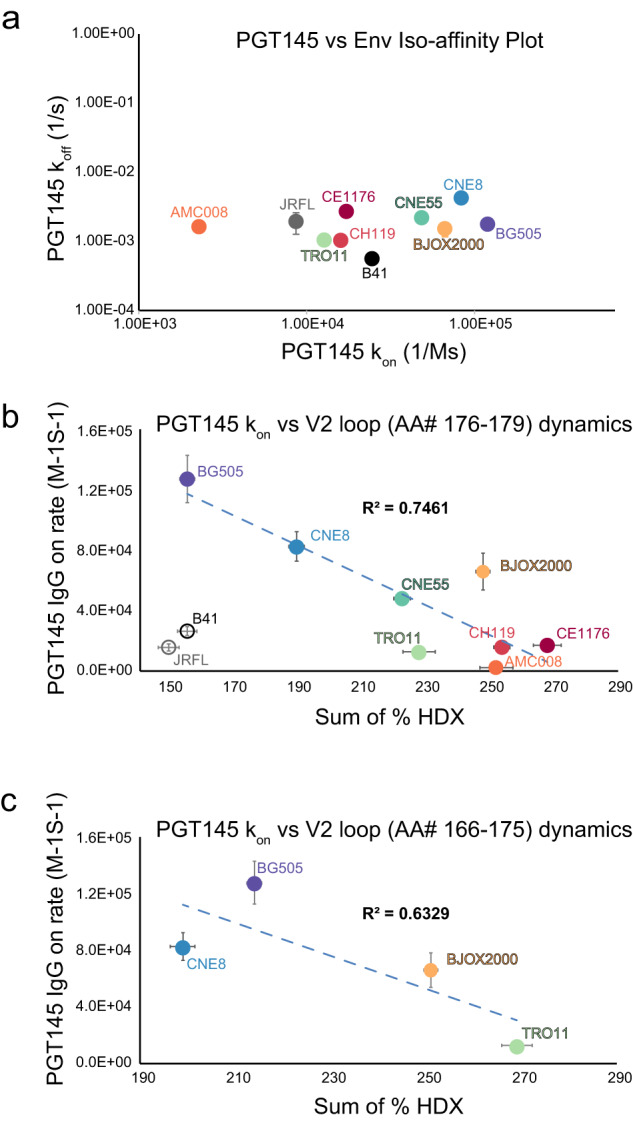
V2 apex-targeting bnAb PGT145 IgG association rates measured by BLI inversely correlate with local dynamics measured by HDX-MS. **a** PGT145 binding to the panel of native-like Env trimers show significant variation in association but only a narrow range of dissociation rates. Data points reflect averages of at least two independent experiments with error bars reporting the standard deviation. Differences in the average PGT145 association rates and epitope dynamics across diverse trimers are correlated in (**b**) and (**c**). The *x*-axis corresponds to the sum of the percent exchange across all time points for the V2 loop peptide FYKL spanning residues 176–179 (**b**) or RDKKQKVYSL spanning residues 166–175 (**c**). The *y*-axis shows the PGT145 association rate measured by BLI. In (**b**) JRFL and B41 are displayed as open circles and not included in the correlation due to differences in epitope sequence. Error bars reflect the square root of the sum of the variances across time for HDX and the standard deviation from at least two independent BLI experiments for the association rates. See also Table S1 and Fig. S1.

Our observation of the highly variable V2 loop ordering in the Env apex and the conformational nature of the epitope targeted by apex antibodies led us to hypothesize that V2 epitope dynamics would impact an antibody’s ability to bind and form a stable complex. Indeed, both homologous V2 loop peptides described above either contain residues that are direct binding contacts to the conformation-specific antibody PGT145 (residues 166–175) or are adjacent to the epitope (residues 176–179). PGT145 IgG binding kinetics were measured using biolayer interferometry (BLI; Figs. 2a, S1, Table S1). Binding affinities, *K*_*D*_*s*, spanned a nearly 50-fold range of magnitudes. The differences in *K*_*D*_*s* were mainly driven by large differences in association rates (*k*_*on*_), and we observed an inverse correlation between binding association rates and V2 loop dynamics at the epitope using the sum of the exchange of peptides spanning residues 166–179 (Fig. 2b, c). The general trend was that isolates with a more ordered, exchange-protected epitope, such as BG505, CNE8, and CNE55 exhibited faster PGT145 IgG association rates in comparison to isolates with more flexible, dynamic epitopes, such as in TRO11, CH119 and CE1176. Outlier isolates B41 and JR-FL both contain differences in sequence at residues that directly contact PGT145 or have been shown to abrogate neutralization potency (168E and 169I respectively, Fig. S2)^23^. We note, there was no apparent trend between epitope dynamics and dissociation rates. Indeed, the PGT145 dissociation rates were fairly similar across all trimer constructs (Fig. 2a, Fig. S1, Table S1). Thus, observed differences in antibody affinity were driven by differences in association rates, and these were significantly impacted by the dynamics of the V2 epitope’s peptide backbone.

In contrast to the observed correlation between local V2 dynamics and antibody binding, no clear correlation was identifiable between binding kinetics and V2 loop length or net charge (Fig. S2). These are properties that in other circumstances have been reported to correlate with apex bnAb neutralization sensitivities (Fig. S2)^24^. Similarly, glycan occupancy at N156 and N160 influences PGT145-mediated neutralization^18^. In our panel of Env trimers, both of these positions were occupied with predominantly high mannose glycoforms (Table S2). In CH119, we were unable to determine the glyocprofile for position N156 due to noisy MS/MS spectra. In B41, only nonglycosylated and complex glycoforms were detected at N156 (Fig. S2b, Table S2). Coverage in this region in JR-FL was lacking in our data, however, we note that other labs have glycoprofiled various JR-FL constructs and found they displayed predominantly high mannose glycans at N156 and complex or high mannose glycans at N160 depending on the construct and purification method used to isolate the JR-FL trimers^25^. Thus differences in glycoforms and glycosite occupancies at these key apex positions do not seem to explain the differences in PGT145 antibody binding trends we observed.

### bnAb PGT145’s binding footprint is highly focused in diverse trimers, despite differences in epitope flexibility

**Fig. 3:**
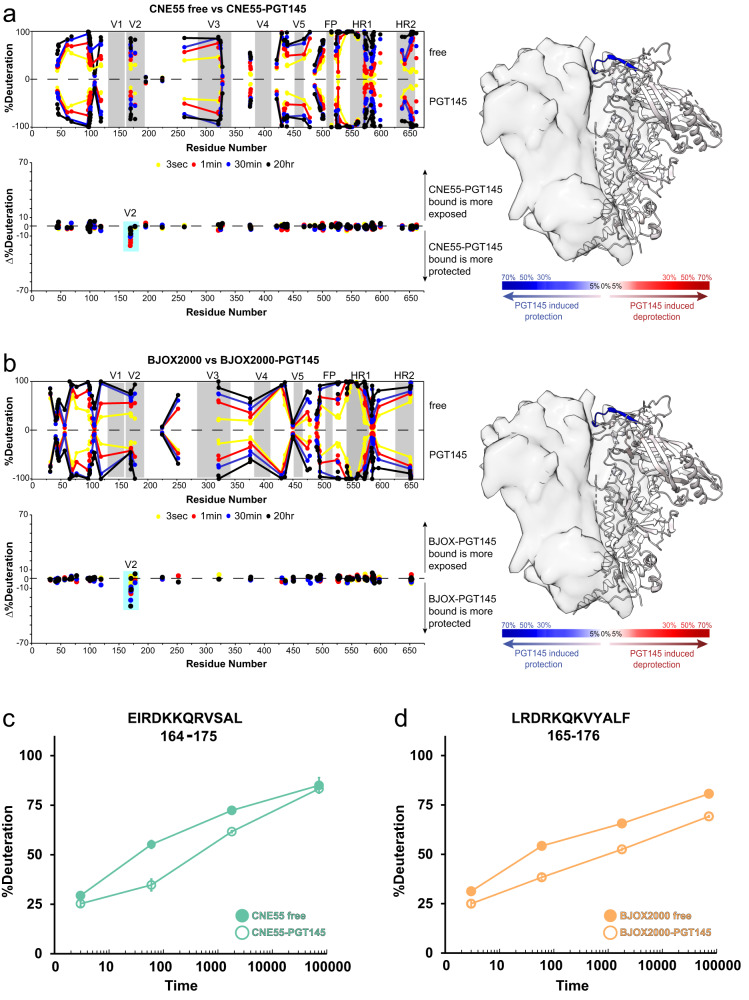
PGT145 binding leads to highly localized protection from deuterium exchange in the V2 loop but does not alter dynamics or structure throughout the rest of the trimers. **a** Profile of local dynamics with and without PGT145 Fab bound to the well-ordered CNE55 trimer shown in “butterfly plot” where each point corresponds to a peptide (mid-point of the peptide segment indicated) at a given time point across the primary sequence. The PGT145 footprint effect is clearly observable in the difference plot shown below. A difference heatmap of changes in deuterium exchange is shown on the right mapped onto pdb 5ACO. Equivalent data for PGT145 Fab binding to BJOX2000 trimer shown in (**b**). Deuterium uptake plots for peptides reflecting PGT145-induced effects shown for CNE55 trimer (**c**) and for BJOX2000 trimer (**d**).

We sought to analyze how the antibody engages with divergent Env trimers such as those with highly dynamic apexes versus those that are more conformationally constrained. We thus applied HDX-MS and single particle cryo-EM analysis to examine the bnAb Fab engaging with a highly dynamic Env trimer, from isolate BJOX2000 and a highly ordered trimer, from isolate CNE55. By quantitatively comparing the deuterium-exchange profiles across the CNE55 and BJOX2000 Env sequences in bound and unbound states, we could identify sites that changed in response to PGT145 binding. The differential HDX-MS results demonstrate that the PGT145 binding footprint is highly localized to the V2 loop (Fig. 3, data S1). The peptides with the largest increase in backbone amide protection contained R166 and K169 (Fig. 3c, d). By contrast, the remainder of peptides throughout both trimers showed negligible change in deuterium exchange protection, including a region near the base of V1 that is part of the bridging sheet and part of the positively charged sink that PGT145 targets at the apex^23^. In this region (residues 112–126, containing K121 and K117; data S1), differences in HDX between the PGT145 bound and unbound states are not detected, suggesting that the epitope-paratope interactions are mediated through sidechains with limited effect on backbone amide dynamics. Previously in a subset of Env trimers, we observed isolate-specific bimodal HDX-MS spectra that are indicative of conformational sampling at localized regions in the Env trimer apex and base^2^. In the BJOX2000 Env trimer, two regions, the CD4 binding loop in gp120 and HR1 in gp41, were identified that exhibit clearly broadened or bimodal isotopic distributions (Fig. S3)^26,27^. CNE55, on the other hand, appears more conformationally uniform, exhibiting only unimodal spectra. We hypothesized that PGT145 binding may shift the conformational equilibrium of Env toward a closed conformational state and eliminate bimodal spectra that may be present in the unbound trimer. Surprisingly, PGT145 binding did not alter the profile of bimodal spectra throughout the BJOX2000 trimer structure (Fig. S3), suggesting antibody binding had minimal long-range allosteric effects throughout the trimer. Thus, in both the highly dynamic BJOX2000 trimer as well as the ordered, conformationally inert CNE55 trimer, PGT145’s structural impact was strictly local and confined to the immediate peptide segment in V2 highlighted in the differential HDX-MS plots (Fig. 3).

### Single-particle cryo-EM structures of PGT145 with divergent Env trimers reveal antibody engagement of N160 glycans across all three protomers

**Fig. 4:**
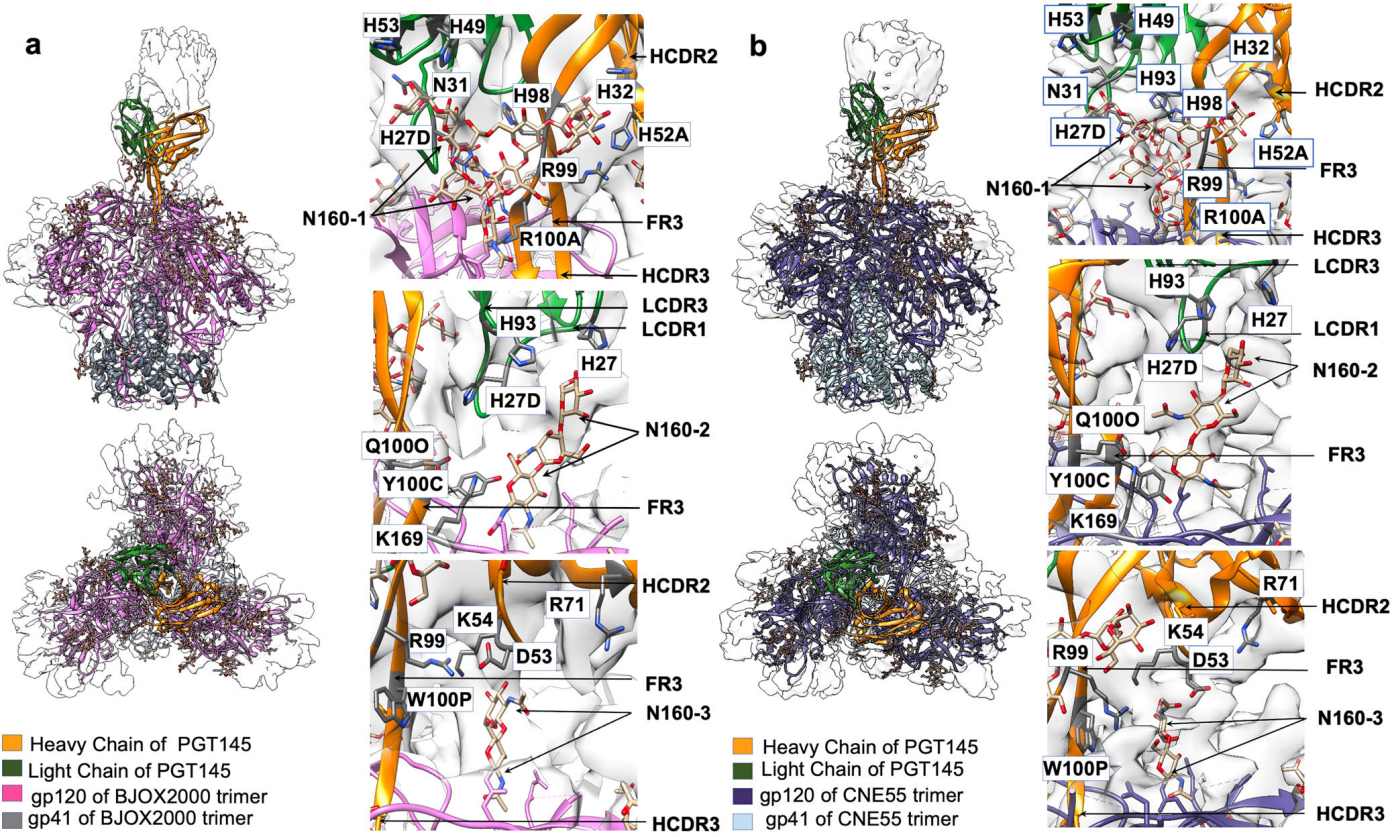
Single-particle cryo-EM reconstructions of two divergent Env native-like trimers with PGT145 Fab reveals engagement of glycans from multiple gp120 protomers is required for PGT145 binding. **a** Side and top view of cryo-EM map with fitted atomic model of BJOX2000 Env+PGT145 Fab. gp120 and gp41 chains are colored magenta and gray. **b** Side and top view of cryo-EM map with fitted atomic model of CNE55 + PGT145. gp120 and gp41 chains are colored purple and sky blue. In all panels, heavy and light chain of the PGT145 Fab are colored orange and green respectively. Interactions of PGT145 with N160 glycans are shown in close-up panels. HCDR: Complementarity determining region of Fab heavy chain, LCDR: Complementarity determining region of Fab light chain, FR: Fab framework region.

To further ascertain the nature of PGT145 recognition of diverse antigens, we obtained single particle cryo-EM structures of the same complexes used in the HDX-MS epitope foot-printing analysis: BJOX2000 + PGT145 Fab and CNE55 + PGT145 Fab. The cryo-EM structures, reconstructed without symmetry imposed, were calculated to a global resolution of 4.87 Å for the BJOX2000 + PGT145 Fab complex and 5.14 Å for the CNE55 + PGT145 complex according to the 0.143 FSC gold standard criterion (Fig. 4, Fig. S4, Table S3). The atomic model of BG505 Env in complex with PGT145 (PDB: 5V8L) was used for initial rigid body fitting into the cryo-EM maps and real space refinement was carried out to optimize map occupancy. The atomic model of BG505 + PGT145 Fab exhibited a good global fit to the cryo-EM maps of both BJOX2000 and CNE55 complexes. RMSD calculations indicate that the SOSIP trimers’ structures are relatively invariant with or without Fab bound among BJOX2000, CNE55, and previously reported BG505 SOSIP structure^15^ (Table S4). However, subtle differences are observed in how the Fab engages with the dynamic BJOX2000 trimer as opposed to the more ordered BG505 trimer. When compared to the BG505 complex, PGT145 nestles into the apex ~4 Å lower in the structure of the BJOX2000 complex along with an ~5 A downward shift in the PGT145 light chain position while engaging the N160 glycan on BJOX2000 (Fig. S5). Due to the limited resolution of the CNE55 complex, we could not confidently make similar comparisons with its structure. Nevertheless, the contacts between Env and Fab, especially the Env contacting HCDR3 loop of PGT145, are maintained across the different complexes.

**Fig. 5:**
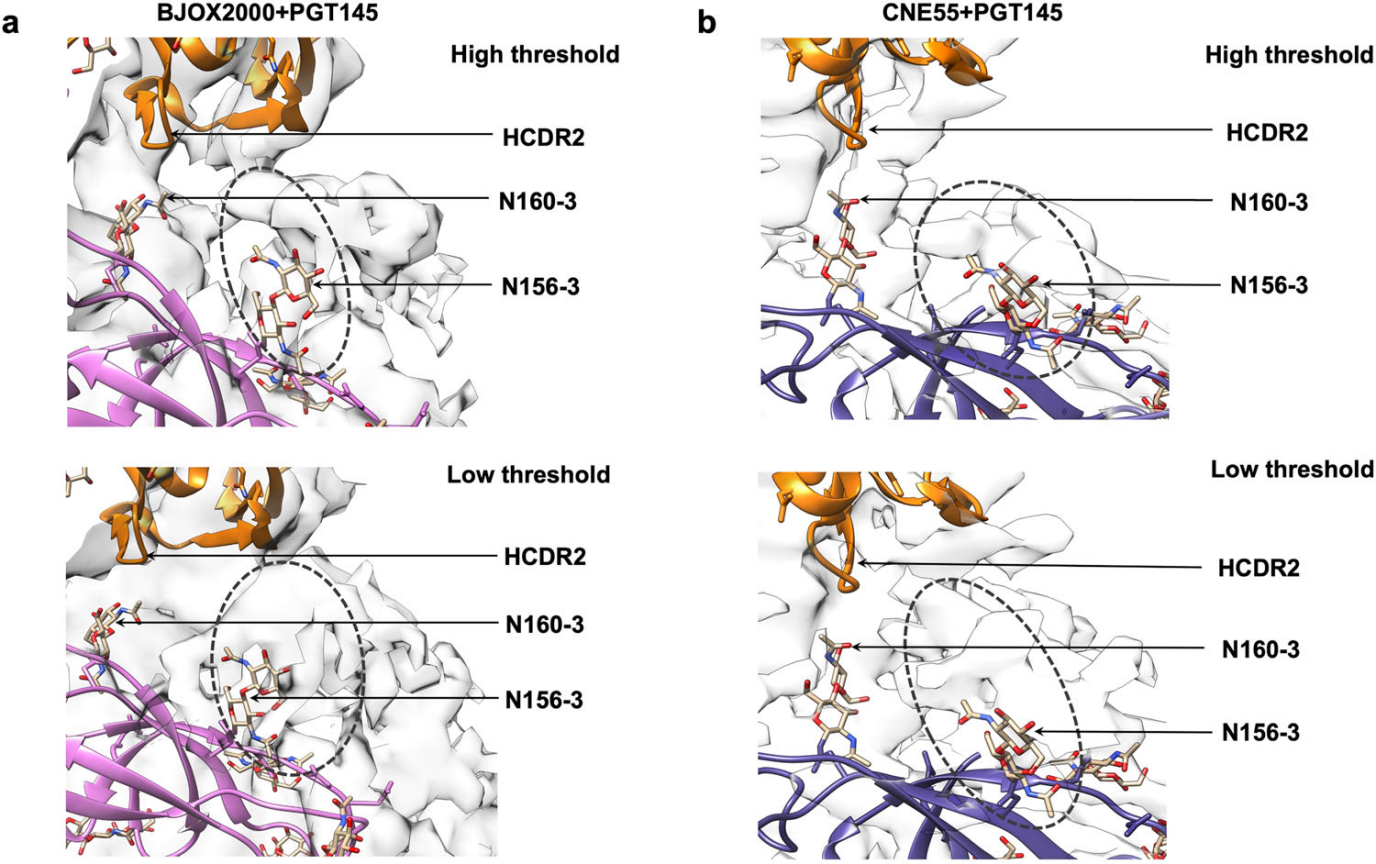
Comparison of N156 Glycan density in the Cryo-EM at low and high threshold. **a** Cryo-EM map of BJOX2000 + PGT145 fitted with atomic model is shown at different thresholds. In case of N156 glycan, interactions are seen only between chain A of Env and HCDR2 of Fab. Clear density connecting Fab and N156 glycan at chain A in the case of BJOX2000 + PGT145 is seen at lower thresholds. In higher threshold also, concerned residues are close enough to interact. **b** Cryo-EM map of CNE55 + PGT145 fitted with an atomic model is shown at different thresholds. In the case of the N156 glycan, interactions are seen only between chain A of Env and HCDR2 of Fab. In the case of CNE55 + PGT145, there is no clear connecting density between N156 glycan and Fab at high threshold, but the concerned residues are close enough for possible interactions. In all panels, density that corresponds to N156 glycan is shown as dotted circle, though glycan chain was not built into density considering the resolution limitation.

Clear density was observed for the N160 glycan in both the BJOX2000 and CNE55 complex maps, with glycan densities in the BJOX2000 + PGT145 Fab complex being more defined due to better local resolution at the Env-Fab interface. Notably, in both maps, N160 glycan density from all three gp120 subunits in a given Env trimer can be seen interacting with the Fab (Fig. 4). This differs from the BG505 + PGT145 Fab structure where only two of the three N160 glycans were reported to be making strong contacts with the Fab (Fig. S6)^15^. In our structures, the density corresponding to N160 glycan from chain 3 (N160-3) interacts with the tip of the Fab’s HCDR2 region (residues G52, D53, K54 Fig. 4) (Table S5). The N160 glycan from chain 2 (N160-2) (Table S5) density shows interaction with the tip of LCDR1 loop of the Fab and with potential interactions with tip of LCDR3 (Fig. 5). The N160 glycan from chain 1 (N160-1) density is expansive and interacts with HCDR1 near N31 residue and HCDR3 near K97-R99. We also resolve contacts between the N160 glycans and framework region 3 that undergo somatic hypermutation (Fig. 4, Fig. S7). Furthermore, the glycan at BJOX2000 N160-2 is shifted ~7.75 Å from the reported BG505 N160-2 glycan position in the complex structures (Figure S5). This may give rise to the downward shift of ~5 Å in the case of the Fab light chain (LCDR1 and LCDR3) of PGT145 in the case of BJOX2000 complexed with PGT145 (Fig. S5). Due to poor local resolution in CNE55 + PGT145, it was not possible to calculate the above-mentioned shifts in LCDR1 and LCDR3 with confidence in this structure.

Notably, in previous structures with PGT145 Fab bound to an Env trimer, N156 glycan density was not well-resolved. Here however, interactions are seen between N156 glycan-3 of Env and HCDR2 of Fab in the case BJOX2000 + PGT145. In the case of CNE55 + PGT145, there is no clear connecting density between N156 glycan and Fab at high threshold, however at lower contour levels of the density map, Fab residues appear to be close enough for possible interactions (Fig. 5). These new maps help to clarify the role that N156 plays in Env recognition by PGT145^15,23^.

The sites of interaction between the Fab and Env regions are similar between BJOX2000 and CNE55 confirming that the Fab binds its target epitope on Env in a conserved manner that is even more dependent on glycan contacts from all three subunits than had been previously appreciated. These interactions appear to form ~30% of the contact surface relative to ~70% for contact with protein surface (Table S6). Moreover, though the overall structures are similar, variations in glycan and protein loop positions are seen in the BJOX2000 complex when compared to the CNE55 and BG505 complex structures. These observations demonstrate that the PGT145 antibody is able to accommodate those differences in local environment in the more dynamic BJOX2000 Env while preserving its common binding contacts.

## Discussion

V1/V2 apex-targeting antibodies, exemplified by PGT145, PG9/16, and the CAP256-VRC26 antibodies, are some of the most potent bnAbs against HIV-1^16,17,21,28,29^. To understand the structural and physical basis for how such bnAbs recognize diverse antigenic targets and conserved features in the midst of variation, it is necessary to examine a given antibody with multiple targets. This study aids in resolving the underlying structural, sequence and dynamic effects responsible for an apex epitope-targeting antibody’s ability to exhibit broad cross-reactivity and neutralization.

Here, cryo-EM and HDX-MS revealed a common mode of interaction and set of contacts with two highly divergent—both in overall sequence and dynamic behavior—Env trimers. As the cryo-EM structures demonstrate, for both the dynamically inert CNE55 trimer as well as the more dynamic BJOX2000 trimer, PGT145 was able to engage N160 from all three protomers, as well as to insert its HCDR3 loop deep into the apex to reach the basic residue sink. As noted, BJOX2000 exhibits greater dynamics across much of the trimer structure including in the apex. It also exhibits local regions with bimodal spectra indicative of transitions between conformations with differences in backbone amide protection. We speculate that the more labile interprotomer interactions in BJOX2000 may permit the PGT145 HCDR3 loop to nestle deeper into the space between V2 loops. Remarkably, even after binding this dynamic target, PGT145 had negligible dynamic impact on even nearby sites that maintained their high level of conformational dynamics. In recent work examining other highly diverse Env trimers, we identified isolate-specific regions throughout Env that sample conformational states on drastically different time scales. Those data indicated that structural elements throughout trimers were not tightly coupled^2^. This may likewise explain why PGT145-induced stabilization of the V1/V2 apex did not appear to propagate through the rest of the trimer. In CNE55, a far more ordered trimer, where one might anticipate the assembly behaves in a unitary, cooperative fashion, the antibody binding likewise had only a local, targeted effect. These findings underscore the exquisitely focused nature of apex antibodies such as PGT145.

Even though PGT145 binding did not appear to order regions outside V2, we hypothesized that isolate-specific differences in V2 loop dynamics could influence PGT145 IgG binding. Indeed, across native-like Env trimers derived from diverse isolates of HIV-1, some of the largest differences in dynamics are found in the V2 loop. This region in the apex has been characterized in different conformations when bound by various Fabs (Fig. S8)^30–34^. While the conformation we and others observe when PGT145 is bound essentially recapitulates the closed, prefusion configuration of the V2 loop, transient dynamic motions may disrupt the quaternary epitope, leading to slower association rates, such as those we observed in some trimers, and this likely imposes a greater entropic cost for binding to more dynamic Env trimers^35,36^. Single-molecule FRET (smFRET) studies examining the effect of PGT145 binding to Env on JR-FL and NL4-3 Env pseudo-typed virions indicated that the populations became biased toward a state that was interpreted to be prefusion Env^3^. Such an effect one might expect would give rise to structural ordering detectable by HDX-MS, however as noted, we did not observe any changes in deuterium exchange beyond the V2 peptide. Taken together, these results suggest that the smFRET observations may reflect a local effect in the apex, as one of the two FRET labels was inserted into the V1 loop near the PGT145 epitope, and shifts in glycan or flexible loop positions may have influenced the disposition of the label. Alternatively, the native membrane-presented Env lacking SOSIP modifications may be more labile than the SOSIP.664 trimers examined in the present study, though the soluble trimers are able to dynamically sample three FRET states as is the case with native Env^37,38^.

How is a bnAb able to bind tightly while having such a minimal impact on the underlying protein substrate? Extensive interactions with conserved glycans (N160 and N156) form a major proportion of the contact surface between PGT145 and Env. Our new cryo-EM structures reveal that glycans from all three gp120 subunits are engaged by PGT145 (Table S3). This includes chain 3, which we find interacts with HCDR2, including forming previously unresolved contacts with residues that undergo somatic hypermutation to produce mature PGT145 (Fig. S7)^15,23^. Loss of a glycan at N156 has also been shown to reduce the neutralization potency of PGT145, suggesting a role in the epitope. Previous studies suggested that the N156 glycan interacts to help stabilize the Env trimer without itself contacting Fab or forming part of the epitope^15,23^. In our PGT45-bound structures, apparent density connecting N156 on chain 3 and PGT145 Fab is seen and appears to contact with HCDR1 where additional sites of somatic hypermutation have been identified, but to date had not been associated with antibody contacts (Fig. S7). By extensively incorporating glycans into the cognate epitope, bnAbs against HIV-1 Env avoid being as reliant on interactions with protein residues, which can vary in accessibility and mutate significantly. While some degree of glycoform heterogeneity is not uncommon, the core portions of most N-linked glycans are largely maintained, and it is these that tend to be recognized by bnAbs^39^. As they are inherently less rigidly ordered in nature than typical globular proteins motifs, glycans also offer some degree of flexibility and variation in coordination by antibodies, as is evident in our structures, and this dampens the effects of underlying protein motions and dynamics.

In summary, we find that Env variation is notably evident in the dynamic nature of the trimers across a panel of diverse HIV-1 isolates. This dynamic variation can be probed in detail using structural mass spectrometry, which reveals both local differences in epitope ordering as well as differences in large-scale conformational switching and dynamic phenotypes. While this study has focused upon one specific bnAb targeting a key epitope, it is conceivable that due to the complex, conformational nature of most bnAb epitopes on Env, similar trends may be observed in those cases as well. Moreover, we anticipate that less affinity matured antibodies found prior to emergence of neutralization breadth will be more profoundly affected by structural and dynamic variation of the nature described in this study. The new structures we report as well as the quantitative characterization of pinpoint ordering of isolate-specific protein dynamics provide a comprehensive portrait of how a bnAb can achieve broad cross-reactivity by exquisitely focused targeting of key conserved features while “threading the needle” of their extremely variable contexts.

## Methods

### Protein expression and purification

SOSIPs were produced and purified as previously described by Verkerke et al. and briefly below^40^. Briefly, Expi293F cells (ThermoFisher Scientific) were transiently transfected at a density of roughly 3 million cells/mL using polyethylenimine (PEI) with plasmids encoding each SOSIP from each respective isolate co-transfected with the furin gene in pcDNA.3.1 at a ratio of 3:1 SOSIP:furin to ensure proteolytic cleavage between gp120 and gp41 subunits during production. After 6 days, the cell supernatants were cleared by centrifugation and filtered through a 0.2 µm vacuum filtration unit and supplemented with protease inhibitors (Roche) and sodium azide to prevent microbial growth. Glycosylated trimers were extracted using Galanthus nivalis lectin (GNL) coupled to agarose beads overnight at 4 °C and washed with 20 mM Tris (pH 7.4), 1 mM EDTA, 1 mM EGTA, 0.02% azide, and 120 mM NaCl; glycoproteins were eluted with 7–10 column volumes of 1 M alphamethyl-mannopyrannoside dissolved in 20 mM Tris (pH 7.4), 1 mM EDTA, 1 mM EGTA, 0.02% Na-azide, and 120 mM NaCl. GNL eluates were concentrated using Amicon ultrafiltration units (nominal molecular mass cutoff of 100 kDa) and buffer exchanged into DEAE low-salt buffer (20 mM Tris [pH 8.0], 100 mM NaCl) before anion-exchange chromatography using a DEAE column. Following 10 min of isocratic flow in 100 mM NaCl, a gradient to 1 M NaCl was initiated and fractions were collected throughout to remove protein aggregates. The DEAE flowthrough was buffer exchanged into 2 M ammonium sulfate– 0.1 M phosphate (pH 7.4) via dialysis and loaded onto a 5 mL HIC HiTrap Phenyl HP column. A step-wise gradient of 2 M–0 M ammonium sulfate in 0.1 M phosphate (pH 7.4) over 90 min was used to separate trimers from dimers and monomers. The early-eluting fractions (containing native-like trimers) were concentrated, and if enough protein was available loaded onto a Superdex S200PG size exclusion chromatography (SEC) column in PBS (20 mM sodium phosphate [pH 7.4], 150 mM sodium chloride, 0.02% sodium azide). Peak fractions were concentrated and characterized by DLS, SDS, BN-PAGE, and negative stain EM (Fig. S9) to ensure homogenous, pure trimer populations immediately prior to HDX and BLI experiments.

PGT145 IgG was produced by transient co-transfection of plasmids containing heavy and light chain fragments at a 1:1 ratio in HEK293F cells. Cultures were allowed to grow for 6 days before harvesting. Secreted IgG was isolated and purified by affinity chromatography using a Hi-Trap Protein A column and eluted using 100 mM Glycine pH 2.0 and neutralized by addition of 1 M Tris pH 8.0. Purity was assessed by SDS-PAGE.

### SDS-PAGE and BN-PAGE

SDS denaturing PAGE and blue native PAGE (BN-PAGE) analyses with precast gels (Novex) were performed to assess the oligomeric species present throughout purification and immediately prior to experiments. 10 µg of protein was loaded per lane for BN-PAGE analysis and 5 µg per lane was loaded for SDS-PAGE analysis.

### Dynamic light scattering (DLS)

Dynamic light scattering (DLS) measurements were performed on a Dynapro Nanostar (Wyatt Technologies). Trimer samples were diluted to 1 mg/ml in PBS and centrifuged at 15,000 × *g* for 20 min prior to loading of 10 μl into a low-volume quartz cuvette. The mean estimated hydrodynamic radius, and polydispersity were generated from 30 acquisitions of 5 s at 20 °C.

### Hydrogen/Deuterium exchange mass spectrometry

5 µgs (42 pmol) per timepoint of each SOSIP construct incubated in deuterated buffer (20 mM PBS, 85% D2O, pH* 7.52) for 3 s, 1 min, 30 min, and 20 h at room temperature. For Env-PGT145 complexes, PGT145 Fab was added at a 4× molar excess to ensure 99% bound Env for 30 min at room temperature prior to incubations in deuterated buffer. The deuteration reaction was stopped via diluting 1:1 in ice-cold quench buffer (200 mM tris(2-chlorethyl) phosphate (TCEP), 8 M urea, 0.2% formic acid) to a final pH of 2.5 and flash frozen in liquid nitrogen followed by storage in −80 °C prior to analysis. Online pepsin digestion was performed and analyzed by LC-MS-IMS utilizing a Waters Synapt G2-Si Q-TOF mass spectrometer as described previously utilizing a 15 min gradient and a home-made HDX cold box that maintains the pepsin digestion at 4 °C and the LC plumbing at 0 °C^40,41^. Pepsin digest eluates from undeuterated sample LC-MS runs were collected, dried by speed vac, incubated in deuteration buffer for 1 h at 65 °C, and quenched as described above to prepare fully deuterated controls. Pepsin digest eluates from undeuterated sample LC-MS runs were also collected, dried by speed vac, resuspended in mobile buffer for peptide identification using nano LC-MS on an Orbitrap Fusion mass spectrometer. A 2 cm trapping column and a 35 cm analytical column were freshly prepared in fused silica (100 μm ID) with 5 μM ReproSil-Pur C18 AQ beads (Dr. Maisch). 8 μL of sample was injected and run using a 60 min linear gradient from 2% to 30% acetonitrile in 0.1% FA, followed by 10 min of 80% acetonitrile. An EThcD method was optimized as follows: ion source: 2.1 kV for positive mode; ion transfer tube temperature: 350 °C; resolution: MS1 = 120,000, MS2 = 30,000; AGC target: MS1 = 2e5, MS2 = 1e5; and injection time: MS1 = 50 ms, MS2 = 60 ms. Orbitrap Fusion data was processed using Byonic (Version 3.8, Protein Metrics Inc.) to obtain a peptide reference list and identify peptic glycopeptides and glycosylation sites. Deuterium uptake analysis was performed with HD-Examiner (Version 2.5, Sierra Analytics) followed by HX-Express v2 for binomial fitting^26,27^. The percent exchange was normalized to the fully deuterated samples. The well characterized BG505 SOSIP was exchanged alongside each new construct as a positive control sample and Internal exchange standards (Pro-Pro-Pro-Ile [PPPI] and Pro-Pro-Pro-Phe [PPPF]) were included in each reaction to control for variations in ambient temperature during the labeling reactions. HDX reaction details, sequence coverage, and repeatability are shown in Fig. S10, Data S1, Table S8.

### Biolayer interferometry

The binding kinetics of the SOSIP constructs against a panel of IgG’s were determined via BLI on an Octet Red system (FortéBio). Anti-human IgG Fc capture biosensors were presoaked in binding buffer (phosphate-buffered saline (PBS pH 7.4) supplemented with 0.1% BSA, 0.005% Tween 20, and 0.02% NaN3) for 10 min. The hydrated tips were then loaded with purified IgG prepared at 8 µg/mL in binding buffer for 80 s. After reaching a stable baseline, antibody-immobilized biosensors were moved into wells containing a twofold dilution series of SOSIP trimer to monitor association for 3 min, then biosensors were moved back into wells containing binding buffer to monitor dissociation for 3 min. Responses were calculated and double referenced against the buffer reference signal and non-specific binding of analyte to biosensor in absence of IgG. Kinetic data were analyzed by using FortéBio Data Analysis 11.0 software and were processed by Savitzky-Golay filtering prior to fitting using a 1:1 binding model. Reported values are averages of data repeated in at least two independent experiments.

### Glycan profiling

To identify the presence of glycans at each glycosite in the V2 loop and assess the relative heterogeneity of glycoforms bottom-up mass spectrometry (MS) was utilized. SOSIPs (0.02mgs) were denatured in a solution containing 25 mM Tris (pH 8.0), 7 M guanidinium chloride (GdnHCl) and 50 mM dithiothreitol (DTT) at 90 °C for 30 min. Reduced cysteines were alkylated by adding fresh iodoacetamide (IAA) to 100 mM and incubating at room temperature for 1 h in the dark. 50 mM excess DTT was then added to quench the remaining IAA. The GndHCl concentration was reduced to 0.6 M by diluting the samples 11-fold with a 10 mM Tris (pH 8.0), 2 mM calcium chloride solution. Samples were then digested using trypsin, and chymotrypsin separately at a ratio of 1:30 (w/w) for 4 h at 37 °C, or a combination of Lys-C and Glu-C at a ratio of 1:30 (w/w). Each SOSIP was digested first by Lys-C for 4 h at 37 °C followed by an overnight digestion of Glu-C 37 °C. All proteases were MS grade (Promega) and the digestion reactions were quenched by the addition of 0.02% formic acid. The digested samples were desalted by Sep-Pak C18 cartridges (Waters) following the manufacturer’s suggested protocol. Glycoform determination was performed via nano LC-MS using an Orbitrap Fusion mass spectrometer (ThermoFisher) as described above in the HDX-MS method section using EThcD and the processing software Byonic (Version 3.8, Protein Metrics Inc.) using a 6 ppm precursor and 10 ppm fragment mass tolerance. Glycopeptides were searched using the N-glycan 309 mammalian database in Protein Metrics PMI-Suite and scored based on the assignment of correct c- and z- fragment ions. The true-positive entities were further validated by the presence of glycan oxonium ions m/z at 204 (HexNAc ions) and 366 (HexNAcHex ions). Glycoforms were categorized as either high mannose: HexNAc(2)Hex(9-5); Hybrid: HexNAc(3)Hex(5-6); or complex (Table S2).

### Quantification and statistical analysis

All of the statistical details of experiments can be found in the Figure legends of each Figure. Hydrogen/deuterium-exchange mass spectrometry experiments were performed in triplicate. Deuterium uptake levels shown in Figs. 1–3 and Data S1 were determined using HD-Examiner (Sierra Analytics) with the error bars reflecting the standard deviation of the technical replicates. Mass spectral analysis to test for the presence of bimodal spectra was performed with HX-Express v2^26,27^.

Biolayer interferometry (BLI) experiments were carried out in at least duplicate. Data represent mean ± the standard deviation as indicated in the Figure legends.

### Cryo-EM sample preparation

Purified Env trimers (CNE55.SOSIP or BJOX2000.SOSIP) were mixed with fragment antigen binding (Fab) of PGT145 antibody in a molar ratio of 1:3 in PBS (phosphate buffer saline) at pH 7.4. The mixture was incubated on ice for 1 h. The final protein concentration of the Env+Fab mixtures were then adjusted to ~0.04 mg/ml using 1XPBS. Lacey grids (Cu) with ultrathin continuous carbon film (400 mesh) (Electron Microscopy Sciences) were glow discharged under vacuum using 25 mA current for 30 s. A 3.0 µl aliquot of sample mixture was applied to the glow discharged grids and blotted for 3–3.5 s before plunging in liquid ethane. Sample vitrification was carried out in a Vitrobot Mark IV unit (FEI Co.) maintained at 4 °C and 100% humidity.

### Cryo-EM data collection

Both samples, the BJOX+PGT145Fab complex and the CNE55 + PGT145 Fab complex, were imaged using an FEI Titan Krios, operating at 300 keV with energy filter (10 eV slit width). Cryo-EM images for BJOX + PGT145 Fab were collected using a K3 direct electron detector (Gatan) in super-resolution mode using Leginon software^42^. Micrographs were collected with a pixel size of 0.86 Å/pixel with each image receiving a dose rate of 8 e-/pixel/sec with 200 ms exposure per frame and 75 frames per image collected. A total of 12358 raw micrographs were collected, among them 3875 micrographs were collected at 25° tilted and rest were collected at 0° tilt using a defocus range of −0.75 to −3 μm.

Cryo-EM images for CNE55 + PGT145 Fab was collected using a K2 Summit direct electron detector (Gatan) in counting mode using Leginon software^42^. Micrographs were collected with a pixel size of 1.35 Å/pixel with each image received a dose rate of 8 e-/pixel/sec with 200 ms exposure per frame and 50 frames per image collected. A total of 2275 raw micrographs were collected, among them 1956 micrographs were collected at 30° tilted and rest were collected at 0° tilt using a defocus range of −0.75 to −3 μm.

### Data processing and structure determination

All data processing steps were performed within the Relion 4.0 software setup^43^. Frame alignment and dose-weighting were performed using MotionCor2^44^ and CTF estimation was done using CTFFIND4^45^.

#### For the BJOX2000+Fab complex data

A total of 4860777 particles from un-tilted data set and 1526177 particles from tilted dataset were picked using the LOG-based automatic particle picking routine within Relion. Good particles were selected through unsupervised 2D classification of the complete particle dataset. Classes which showed clear SOSIP bound to 3 Fabs were selected. The published structure of BG505+PGT145Fab (EMD-8643) was low-pass filtered to 60 Å and used as initial model. 3D reconstruction of particle set was performed with no symmetry imposed (C1) to generate a 3D density map. The particles were then subjected to 3D classification using the initial reconstruction as starting model. 3D classes were selected containing total particle number of 667012. These were then used for 3D refinement to generate a 3D density map, which after post-processing in Relion resulted in a 4.9 Å structure using the ‘gold-standard’ FSC cutoff of 0.143.

The atomic model of BG505 SOSIP Trimer in complex with PGT145 Fab (PDB ID: 5V8L)^15^ was rigid body fitted into the 4.9 Å resolution map using UCSF Chimera^46^. In the cryo-EM map, HCDR3 of PGT145-Fab was well resolved, along with well-resolved gp41 alpha helix. The cryo-EM map was further sharpened with input model of BG505 SOSIP trimer in complex with PGT145 (PDB ID: 5V8L) using Local Anisotropic sharpening in the Phenix software^47^. The model was then refined using Real space refinement in the Phenix package using secondary structure restraints. The resulting model was iteratively fixed and refined in Coot^48^ employing Ramachandran constraints. Glycan at chain C had to be shifted manually by ~5 Å to fit the clear N160 glycan density.

#### For the CNE55+Fab complex data

A total of 1,602,666 particles from tilted data set and 238,605 particles from untilted data set were picked using the LOG-based automatic particle picking routine. Particle classes showing clear images of SOSIP bound to Fab were selected through unsupervised 2D classification of the total particle dataset. The CNE55 dataset suffered from preferred orientation bias with a significantly large number of classes showing only top or bottom views of the Env trimer complex. Thus, using the combined tilted and un-tilted dataset, multiple rounds of 2D classification were performed to separate out as many distinct orientational views as possible. A total of 1015980 particles were selected finally from 2D classification for 3D reconstruction. The published structure of BG505+PGT145Fab (EMD-8643) was low-pass filtered to 60 Å and used as initial model. Similar to the BJOX2000+Fab structure, 3D reconstruction of particle set was performed with no symmetry imposed (C1) to generate a 3D density map followed by 3-D classification. 2 out of 4 generated 3D classes were selected containing total particles 527385. These were then used for 3D refinement to generate a 3D density map with global resolution of 6.75 Å. Subsequent CTF refinement and post-processing in Relion resulted in a 5.14 Å structure using the ‘gold standard’ FSC cut-off of 0.143.

The atomic model of BG505 SOSIP trimer in complex with PGT145 (PDB ID: 5V8L)^15^ was rigid body fitted into the 5.14 Å resolution map using UCSF Chimera^46^. The cryo-EM map was sharpened to enhance feature in the maps similar to the BJOX2000+Fab complex map as above. The fitted atomic model was then refined using real space refinement in the Phenix package using secondary structure restraints^47^.

The data collection and refinement parameters along with final model statistics are given in the Supplementary Table S3. Local map resolution was estimated using ResMap software (Fig. S4)^49^.

## Supplementary Information

Supplementary information

Data S1

## Data Availability

Cryo-electron microscopy data presented in this study has been submitted to the Electron Microscopy Data Bank (EMDB) under accession codes EMD-36641 and EMD-36649. The fitted coordinates have also been deposited in the RCSB Protein Data Bank (PDB) under accession codes 8JTD and 8JTM. Mass spectrometry data will be made available upon request.
