# Supplementary material for: An HIV-1 broadly neutralizing antibody overcomes structural and dynamic variation through highly focused epitope targeting
Source: Npj Viruses. 2023 Oct 5;1:2. doi: 10.1038/s44298-023-00002-4 (PMC11041648; doi:10.1038/s44298-023-00002-4)
Supplement: Supplementary file 1 — Supplementary information [file 44298_2023_2_MOESM1_ESM.pdf]

SUPPLEMENTARY INFORMATION

for

An HIV-1 Broadly Neutralizing Antibody Overcomes Structural and Dynamic Variation through Highly Focused Epitope Targeting

Edgar A. Hodge[1], Ananya Chatterjee[3], Chengbo Chen[1,2], Gajendra S. Naika[1],

Mint Laohajaratsang[1], David Montefiori[4], Vidya Mangala Prasad[1,3,5,*], Kelly K. Lee[1,2,*]

Supplementary Tables S1-S7

Supplementary Figures S1-S10

Supplementary Tables:

Table S1. Isolate-specific differences in binding Kinetics of PGT145 IgG measured by biolayer interferometry (BLI). BLI-determined binding affinities (K_D), binding association rates (k_{on}), and dissociation rates (k_{off}) for PGT145 IgG are shown. Values reported are the average of at least two independent experiments with standard deviations shown.

**PGT145 Binding Kinetics**			
**SOSIP**	**PGT145 K_D (nM)**	**PGT145 K_{on} (1/Ms)**	**PGT145 k_{off} (1/s)**
**AMC008**	735.4 ± 246.9	2.29E+03 ± 4.41E+02	1.63E-03 ± 2.42E-04
**B41**	23.4 ± 0.9	2.46E+04 ± 2.47E+03	5.60E-04 ± 3.35E-05
**CNE8**	50.8 ± 9.9	8.34E+04 ± 9.86E+03	4.19E-03 ± 3.27E-04
**TRO11**	80.75 ± 14.4	1.29E+04 ± 1.21E+03	1.03E-03 ± 8.75E-05
**BJOX**	27.7 ± 9.3	6.67E+04 ± 1.24E+04	1.52E-03 ± 3.43E-04
**BG505**	16.0 ± 3.9	1.20E+05 ± 4.07E+04	1.77E-03 ± 1.54E-04
**CE1176**	156.5 ± 19.1	1.73E+04 ± 2.47E+02	2.71E-03 ± 3.80E-04
**CNE55**	45.35 ± 1.1	4.86E+04 ± 7.78E+02	2.20E-03 ± 8.98E-05
**JRFL**	223.33 ± 71.5	8.66E+03 ± 1.15E+03	1.94E-03 ± 6.69E-04
**CH119**	64.55 ± 6.2	1.60E+04 ± 3.68E+02	1.03E-03 ± 7.45E-05

Table S2. N156 and N160 Glycoprofiles for panel of native-like Env trimers. Each Asn (N) residue at residues 156 and 160 in the V2 loop with the NxS/T motif indicating a glycosite is listed with the glycoforms identified via MS/MS using EThcD. Regions with no coverage are listed as NC, and ND indicates areas where the glycoform was not determined (for example low quality/scoring Ms/Ms spectra). Glycoforms were categorized as either high mannose: HexNAc(2)Hex(9-5) (green) or complex (red). Abbreviations and glycan classifications used are according to Essentials of Glycobiology, 3rd ed. HexNAc: N-acetylhexosamines; Hex: Hexose; Fuc: L-Fucose; NeuAc: N-acetyl neuraminic acid.

	**BG505**		**CE1176**		**B41**	
**Asn (N) Residue number**	**156**	**160**	**156**	**160**	**156**	**160**
	HexNAc(2)Hex(9)	HexNAc(2) Hex(9)	HexNAc (2) Hex(9)	HexNAc(2) Hex(7)	nonglycosylated	nonglycosylated
	HexNAc(2)Hex(8)	HexNAc(2) Hex(8)	HexNAc (2) Hex(7)	HexNAc(2) Hex(5)	HexNAc(5) Hex(6) Fuc(2)NeuAc(3	HexNAc(4)Hex(5) Fuc(2)NeuAc(2)
	HexNAc(2)Hex(7)	HexNAc(2) Hex(7)		HexNAc(2) Hex(4)		HexNAc(2)Hex(6)
	HexNAc(2)Hex(6)	HexNAc(2) Hex(6)		HexNAc(2) Hex(3)		HexNAc(2)Hex(7)
	HexNAc(3)Hex(3) Fuc(1)	HexNAc(2) Hex(5)				HexNAc(2)Hex(8)
	HexNAc(5)Hex(3) Fuc(1)					HexNAc(2)Hex(5)
	HexNAc(4)Hex(3) Fuc(1)					HexNAc(2)Hex(9)

	**AMC008**		**JRFL**		**CNE55**	
**Asn (N) Residue number**	**156**	**160**	**156**	**160**	**156**	**160**
	HexNAc(2) Hex(9)	nonglycosylated	NC	NC	HexNAc(2) Hex(9)	non-glycosylated
	HexNAc(2) Hex(8)	HexNAc(2) Hex(9)			HexNAc(2) Hex(8)	HexNAc(2)Hex(6)
		HexNAc(2) Hex(8)				HexNAc(2)Hex(5)
		HexNAc(2) Hex(7)				HexNAc(2)Hex(7)
		HexNAc(2) Hex(6)				
		HexNAc(2) Hex(5)				
		HexNAc(2) Hex(4)				
		HexNAc(5) Hex(6)				
		HexNAc(3) Hex(3)Fuc(1)				
		HexNAc(3) Hex(4)Fuc(1)				

	**BJOX2000**		**TRO11**		**CH119**	
**Asn (N)**	**156**	**160**	**156**	**160**	**156**	**160**

Residue number						
	non-glycosylated	non-glycosylated	non-glycosylated	non-glycosylated	nonglycosylated	HexNAc(2)Hex(8)
	HexNAc(2)Hex(7)	HexNAc(2) Hex(8)	HexNAc(9)	HexNAc(2)	not determined	HexNAc(2)Hex(5)
		HexNAc(2) Hex(7)	HexNAc(7)	HexNAc(8)		HexNAc(5)Hex(3) Fuc(1)
		HexNAc(2) Hex(6)		HexNAc(9)		
		HexNAc(2) Hex(5)				
	**CNE8**					
**Asn (N) Residue number**	**156**	**160**				
	nonglycosylated	HexNAc(2) Hex(9)				
	HexNAc(2)Hex(8)	HexNAc(2) Hex(8)				
	HexNAc(2)Hex(7)	HexNAc(2) Hex(7)				
	HexNAc(2)Hex(6)	HexNAc(2) Hex(5)				
	HexNAc(6) Hex(6)NeuAc(3)	HexNAc(5)Hex(5) Fuc(1)NeuAc(1)				
		HexNAc(6)Hex(7) Fuc(1)				
		HexNAc(5)Hex(5) Fuc(2)NeuAc(2)				

Table S3. Cryo-EM Data collection, processing, refinement and validation statistics.

	**Single particle Cryo-EM map of BJOX2000+PGT145**	**Single particle Cryo-EM map of CNE55+PGT145**
**Data collection and processing**		
Magnification	×105,000	×105,000
Voltage (KV)	300	300
Electron exposure (e–/Å2)	1.44	0.88
Defocus range (μm)	0.75-3 μm	0.75-3 μm
Pixel size (Å)	0.86	1.35
Symmetry imposed	C1	C1
Initial particle images (no.)	4860777(untilted), 1526177(tilted)	238605(untilted) , 1602666(tilted)
Final particle images (no.)	667012	527385
FSC threshold Map resolution range (Å)	4.9	5.14
**Model refinement and validation statistics**		
Initial model used (PDB code)	5V8L	5V8L
Clashscore, all atoms	19.78	20.37
Poor rotamers	0	0
Favored rotamers	100	100
Ramachandran outliers	0.05	0.0
Ramachandran favored	96.14	96.71

Table S4: RMSD Calculation of liganded BG505 SOSIP and unliganded BG505 SOSIP with BJOX2000 and CNE55 complexed with PGT145. Measurements were calculated between 4ZMJ trimer (a BG505 unliganded SOSIP structure) and the BJOX2000+PGT145 or CNE55+PGT145 coordinates with Fab chains deleted, or between 5V8L (BG505+PGT145 Fab) and our PGT145 Fab-bound BJOX2000 or CNE55 structures.

**Envelope Trimer Pairs**	**RMSD calculation (Å)**
BG505 SOSIP (4ZMJ) and BJOX2000	0.956 Å
BG505 SOSIP (4ZMJ) and CNE55	0.939Å
BG505+PGT145 (5V8L) and BJOX2000+PGT145	0.496Å
BG505+PGT145 (5V8L) and CNE55+PGT145	0.392 Å

Table S5: Chain IDs across different PDB models of Env structures compared and denoted as chains 1,2 &3 in this study.

	Env chain for N160 glycan 1 (N160-1)	Env chain for N160 glycan 2 (N160-2)	Env chain for N160 glycan 3 (N160-3)
BJOX2000	D	C	A
CNE55	D	C	A
BG505	D	C	A
AMC011	A	E	C

Table S6. EM structure contact area calculation.

The contact area between Fab and the trimer were calculated using the solvent accessible area (SA) of BJOX and CNE55 atomic models. The total SA of the Env trimers with and without N160 glycan, env-Fab complex, env-Fab complex without glycans and only Fab is calculated and provided in the above table.

For calculating the contact area at Env-Fab interface:

[(Total SA of trimer + Total SA of Fab) – Total SA for complex] / 2

a	Total trimer SA (Å^2)	Total trimer SA w/o N160 glycans (Å^2)	Total complex SA (Å^2)	Total complex SA w/o N160 glycans (Å^2)	Total SA for Fab (Å^2)
BJOX2000	94113.4	92603.4	104784	104377	14307.2
CNE55	88857.2	87264.4	100022	99489.4	14307.2

b	Total contact area (Å^2)	Contact area w/o N160 glycan (Å^2)	Contact area of N160 glycan (Å^2)
BJOX2000	1818.3	1266.8	551.5
CNE55	1571.2	1041.1	530.1

Table S7. HDX reaction conditions and statistics for mapping epitope foot-printing.

Dataset	CNE55.664	CNE55-PGT145
HDX reaction details	85% D2O buffer, pH* 7.466, labeled at RT (22.0°C), Quenched at pH 2.502 in 200mM TCEP, 8M urea, 0.2%FA	85% D2O buffer, pH* 7.466, labeled at RT (22.0°C), Quenched at pH 2.502 in 200mM TCEP, 8M urea, 0.2%FA
HDX time course	3 sec, 1 min, 30 min, 20 hrs	3 sec, 1 min, 30 min, 20 hrs
HDX controls	PPPI, PPPF	PPPI, PPPF
Avg Back-exchange	12.7%	13.4%
Number of peptides	184	184
Sequence coverage	61.5%	61.5%
Average peptide length/redundancy	13.4 residue length/ 3.8 redundancy	13.4 residue length/ 3.8 redundancy
Replicates (biological or technical)	two technical replicates	two technical replicates
Repeatability	Stddev 1.04%	Stddev 0.93%

Dataset	BJOX2000.664	BJOX2000-PGT145
HDX reaction details	85% D2O buffer, pH* 7.466, labeled at RT (22.0°C), Quenched at pH 2.502 in 200mM TCEP, 8M urea, 0.2%FA	85% D2O buffer, pH* 7.466, labeled at RT (22.0°C), Quenched at pH 2.502 in 200mM TCEP, 8M urea, 0.2%FA
HDX time course	3 sec, 1 min, 30 min, 20 hrs	3 sec, 1 min, 30 min, 20 hrs
HDX controls	PPPI, PPPF	PPPI, PPPF
Back-exchange	9.0	9.0
Number of peptides	127	127
Sequence coverage	57.8	57.8
Average peptide length/redundancy	12.8 residue length/ 2.6 redundancy	12.8 residue length/ 2.6 redundancy
Replicates (biological or technical)	two technical replicates	two technical replicates
Repeatability	Stddev of 1.37%	Stddev of 1.32%

Supplementary Figures:

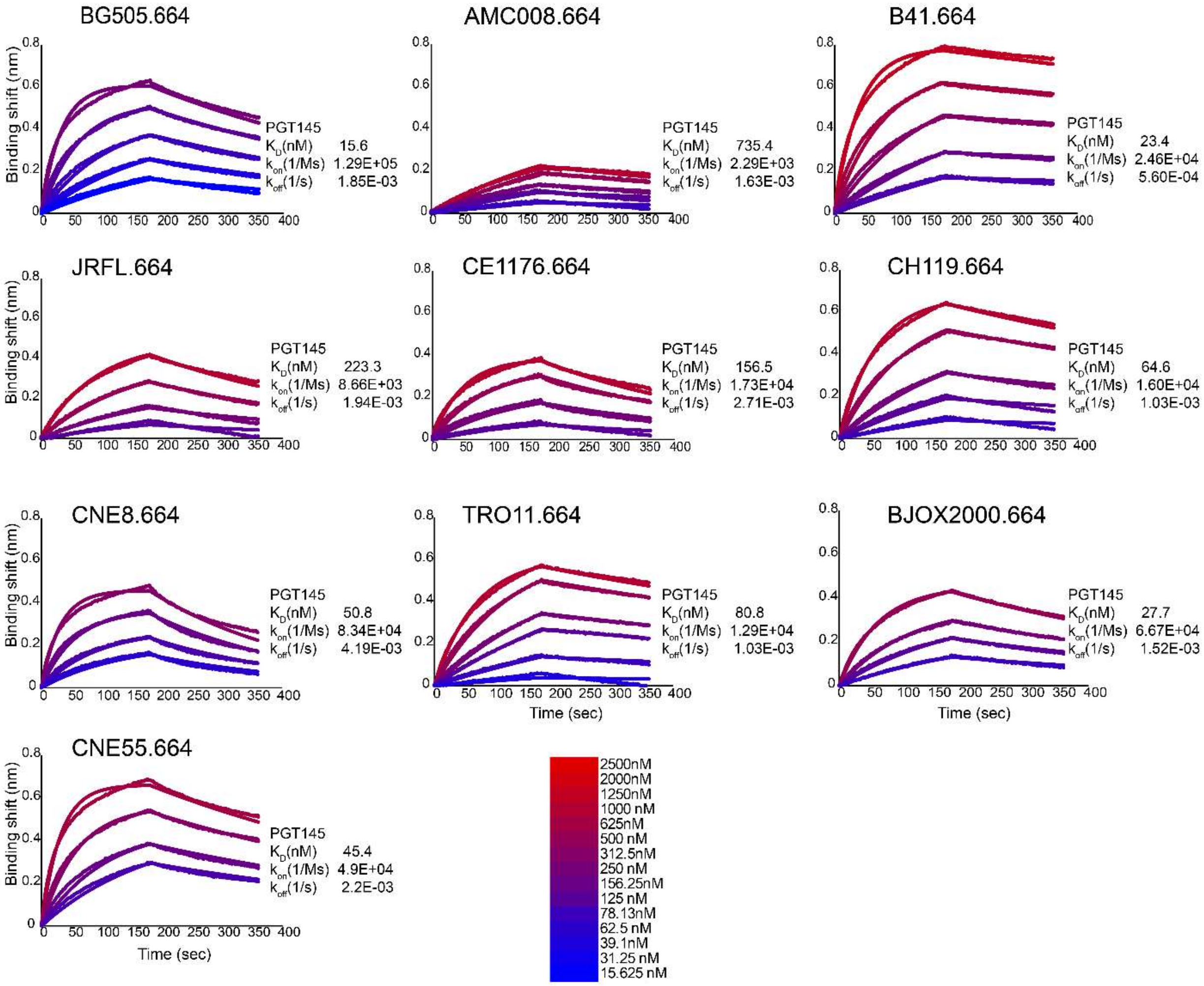

Figure S1. PGT145 IgG binding kinetics to Env trimers derived from a panel of HIV-1 isolates. Representative BLI sensorgrams of PGT145 binding to serially diluted SOSIPs derived from different HIV isolates are shown. SOSIP analyte concentrations are shown according to the legend using a red to blue color gradient.

a

```
                                    B        C       C' hypervariable region  D
HXB2                            156 NCSFNISTSIRGKVQKEYAFFYKLDIIPIDND--------TTSYKLTSCNTS 199
BG505     (clade A)                 NCSFNMTTELRDKKQKVYSLFYRLDVVQINENQGNRSNNSNKEYRLINCNTS
CNE55     (clade AE)                NCTFNMTTEIRDKKQRVSALFYKLDIVPIDDSKN------NSEYRLINCNTS
B41       (clade B)                 NCSFNVTTSIRDKIKKEYALFYKLDVVPLENKNNI-NNTNITNYRLINCNTS
AMC008    (Clade B)                 NCSFNVTTSIRDKVQKEYALFYKLDIVPIDDDN------NTSNYRLINCNTS
JRFL      (clade B)                 NCSFNITTSIRDEVQKEYALFYKLDVVPIDNN--------NTSYRLISCDTS
TRO11     (clade B)                 NCSFNITAGIRDKVKKEYALFYKLDVVPIEEDKD----TNKTTYRLRSCNTS
CH119     (CRF07_BC)                NCSFNATTVVRDRQQKVYALFYRLDIVPLTEK--NSSENSSKYYRLINCNTS
CNE8      (clade C)                 NCSFNTTTELRDKKQNVYALFYKLDIVPIN---N------NSEYRLINCNTS
CE1176    (clade C)                 NCSFNATTEIKDKKKNEYALFYKLDIVPLNN--------SSGKYRLINCNTS
BJOX2000  (CFR07)                   NCSFNATTELRDRKQKVYALFYKLDIVPLNEK--NSS-----EYRLINCNTS
```

■: residues that interact directly w/ PGT145 paratope

■: residues that effect neutralization sensitivity

b

Isolate	V2 length	Net charge	N156 glycan	N160 glycan
BG505	46	+3	H	H
CE1176	38	+2	H	H
B41	45	+3	C	H
AMC008	40	-1	H	H
JRFL	38	-2	NC	NC
CNE55	40	+2	H	H
BJOX	38	+2	H	H
TRO11	41	+2	H	H
CH119	43	+3	ND	H
CNE8	37	+1	H	H

H: high mannose
C: complex
ND: not determined
NC: no coverage

c

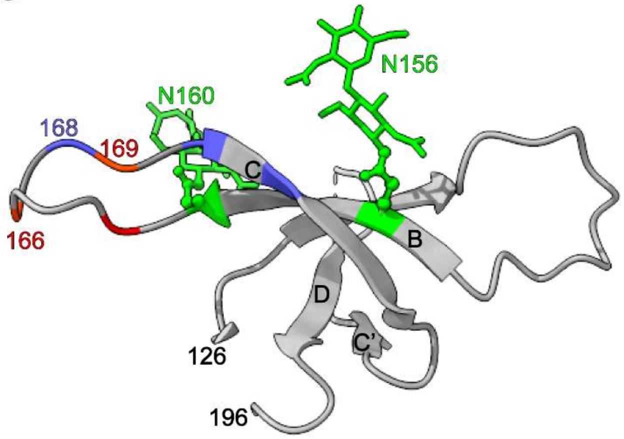

Figure S2. V1/V2 characteristics across panel of native-like Env trimers. These properties could impact bnAb recognition of the V2 region of trimers, however we do not observe correlations between these and PGT145 binding. The PGT145 antibody target the V2 loop trimer apex hole and the positively charged C-strand of Env across diverse HIV isolates. The sequences of the V2 loops across diverse isolates of HIV are aligned **(a)**. Residues that directly interact with PGT145 (residues 162, 166 and 169) are colored red, residues that have been

shown to influence neutralization sensitivity are colored blue, and glycans at N156 and N160 are highlighted in green. The table in **(b)** displays the V2 loop length, net charge, and predominate glycoform at N156 and N160. The structure of the V1/V2 4 stranded beta-barrel (from BG505 structure PDB 4VTP) is shown in ribbon representation in **(c)**. Antibody binding contacts are colored as in figure a, and the base two HexNac sugars at N156 and N160 are shown in stick representation. See also Table S2.

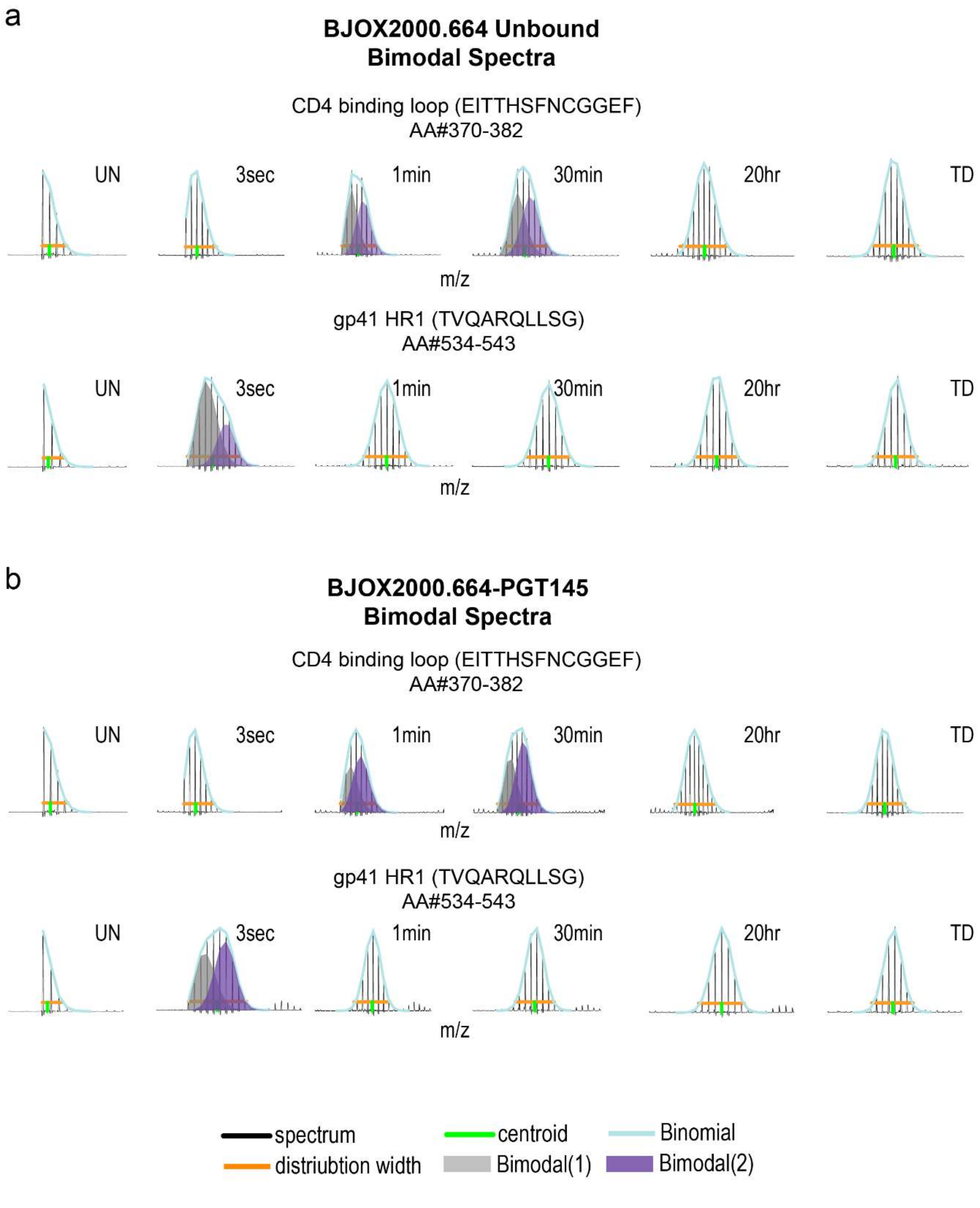

Figure S3. PGT145 binding does not abrogate conformational sampling throughout the BJOX2000 trimer structure. Two representative peptides in BJOX2000.664 unbound and BJOX2000 bound to PGT145 exhibiting unusually broad isotopic distributions were binomially fit to two populations (**a, b** respectively). Each panel displays the spectra from a deuteration

reaction time point. The lighter in mass/more protected population is shaded in gray, and the heavier in mass/faster exchanging population is shaded in purple. The legend describing the centroid, peak envelope, and binomial fit are displayed in the bottom center of the figure.

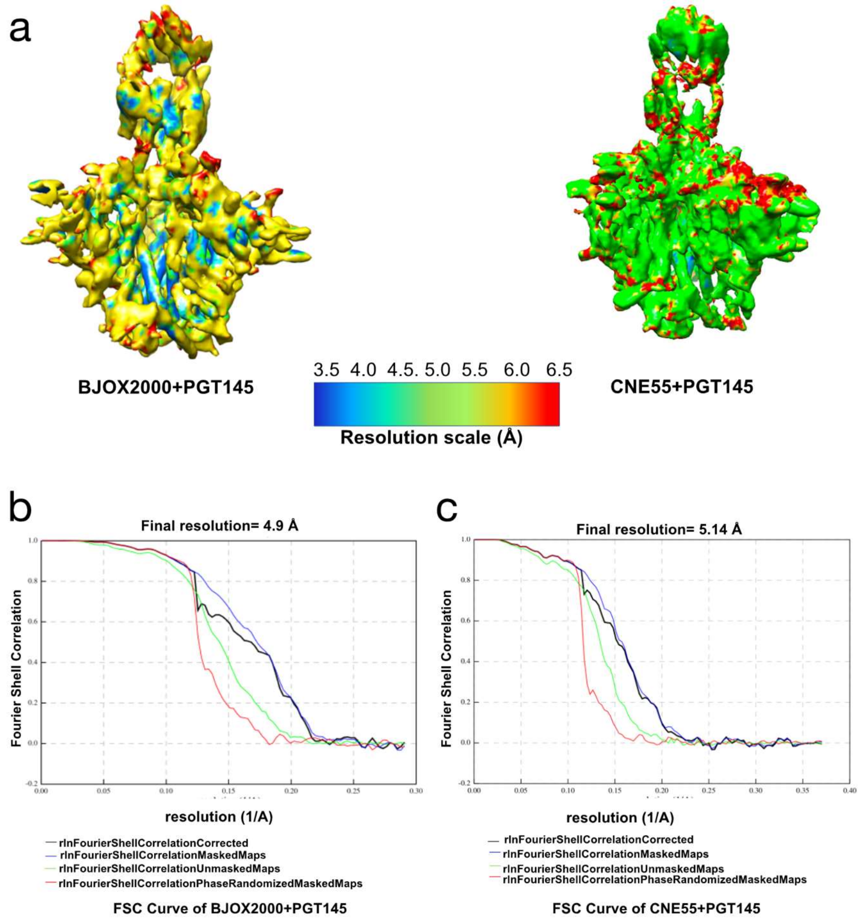

Figure S4. A. Local resolution map evaluation of BJOX-PGT145 and CNE55-145 made using ResMap. (a) Cryo EM structure of BJOX2000 and CNE55 in complex with PGT145 solved at 4.9 Å and 5.14 Å respectively, with a local resolution between 3.5 to 6.5 Å. **(b,c)** FSC curves of BJOX+PGT145 and CNE55+PGT145. The cryo-EM structures, without any symmetry imposed, were calculated to a global resolution of 4.9 Å for the BJOX2000+PGT145 Fab complex and 5.14 Å for the CNE55+PGT145 complex according to the 0.143 FSC gold standard criterion.

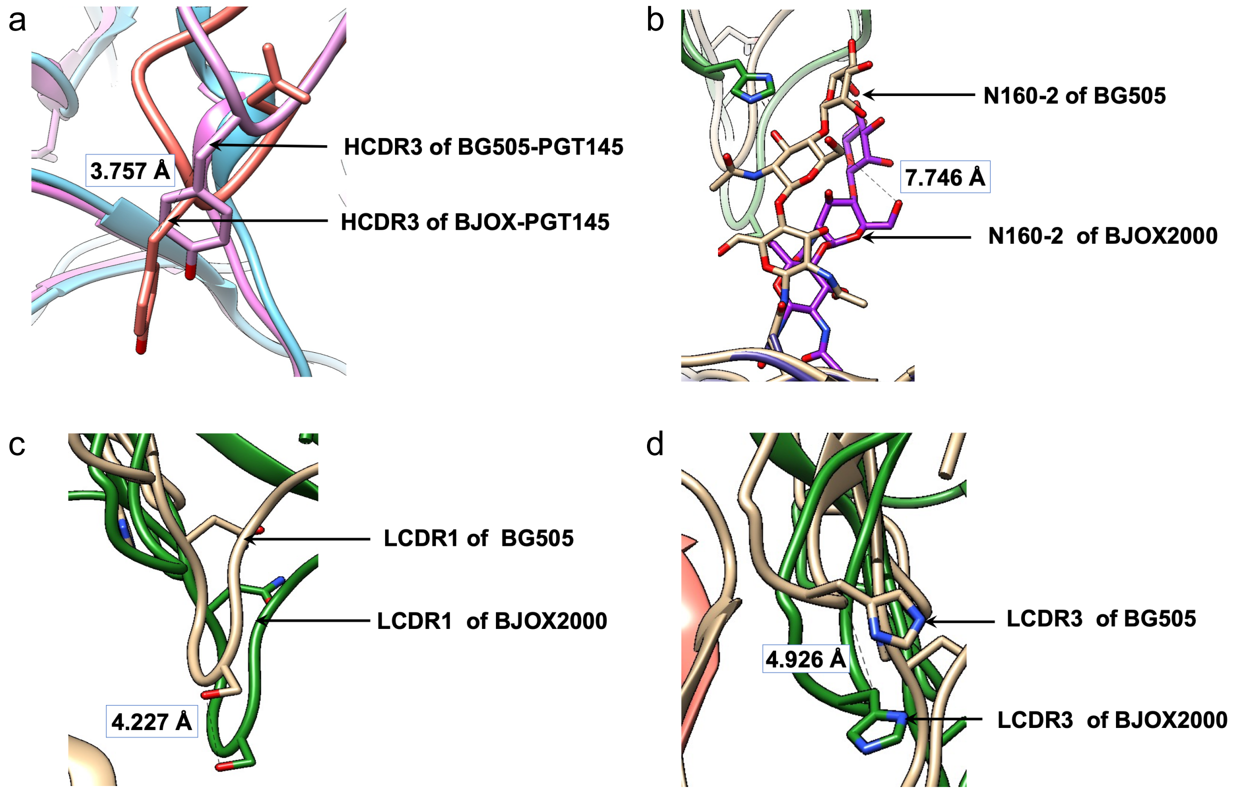

Figure S5. Comparison of the position of Fab structural elements between BJOX-PGT145 and BG505-PGT145 complex structures. (a) HCDR3 of BJOX2000+PGT145 Fab complex is shifted ~4 Å downwards compared to that in BG505+PGT145 complex structure. **(b)** N160 glycan in chain 2 of BJOX2000+PGT145 complex structure is shifted approximately 7.5 Å compared to the same N160 glycan in BG505+PGT145 complex. **(c,d)** Downward shift of ~5 Å observed in Fab light chain regions (LCDR1 and LCDR3) of BJOX2000+PGT145 complex structure compared to the BG505+PGT145 complex.

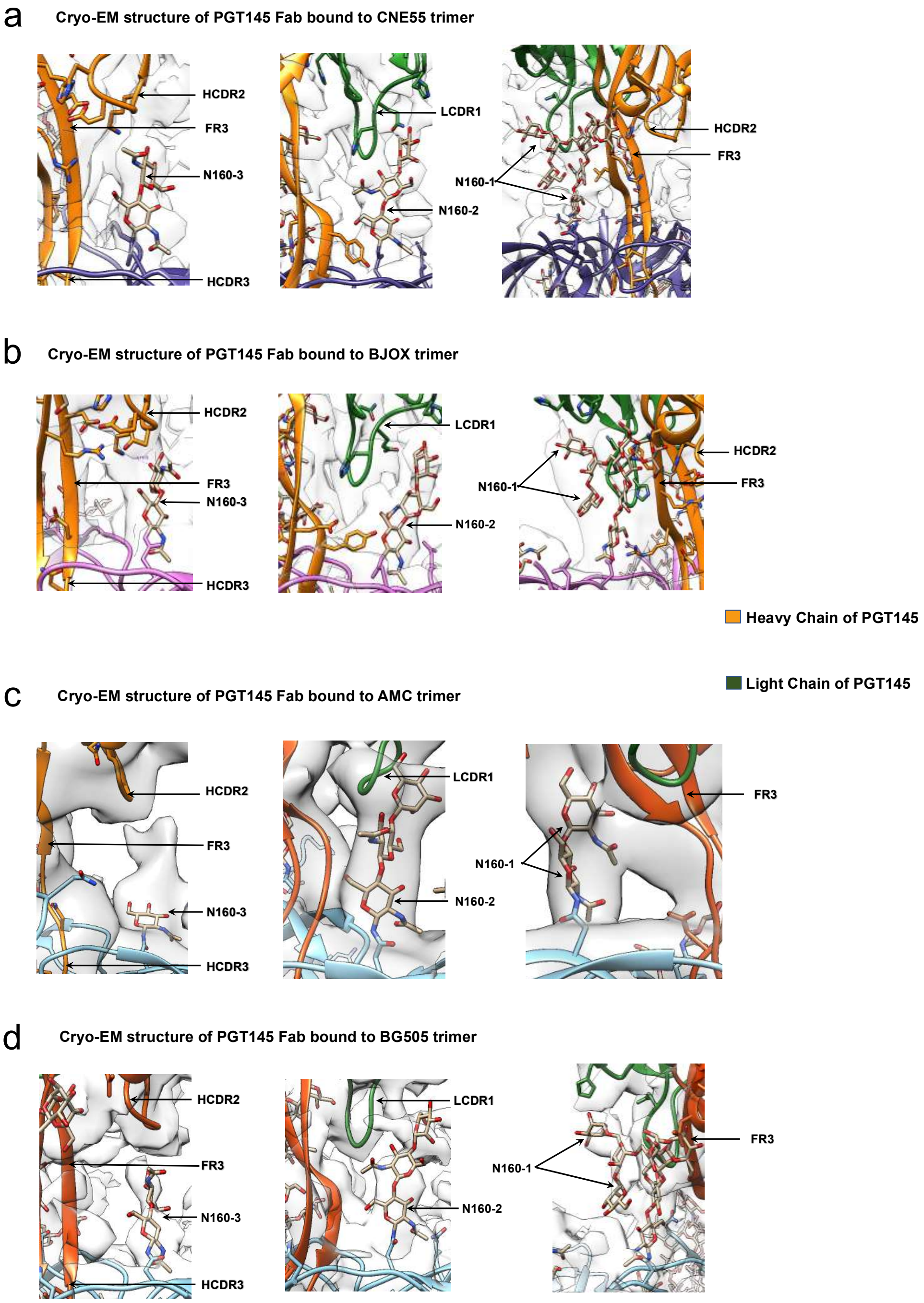

Figure S6. Comparison of N160 glycan density among Cryo-EM structures of BG505, BJOX2000, CNE55 and AMC011 trimers. (a) shows the zoomed in Cryo-EM structure of CNE55 bound to PGT145, **(b)** the structure of BJOX2000 bound to PGT145, **(c)** the structure of

AMC011 bound to PGT145 (EMD-9378), and **(d)** the structure of BG505 bound to PGT145 (EMD-8643).

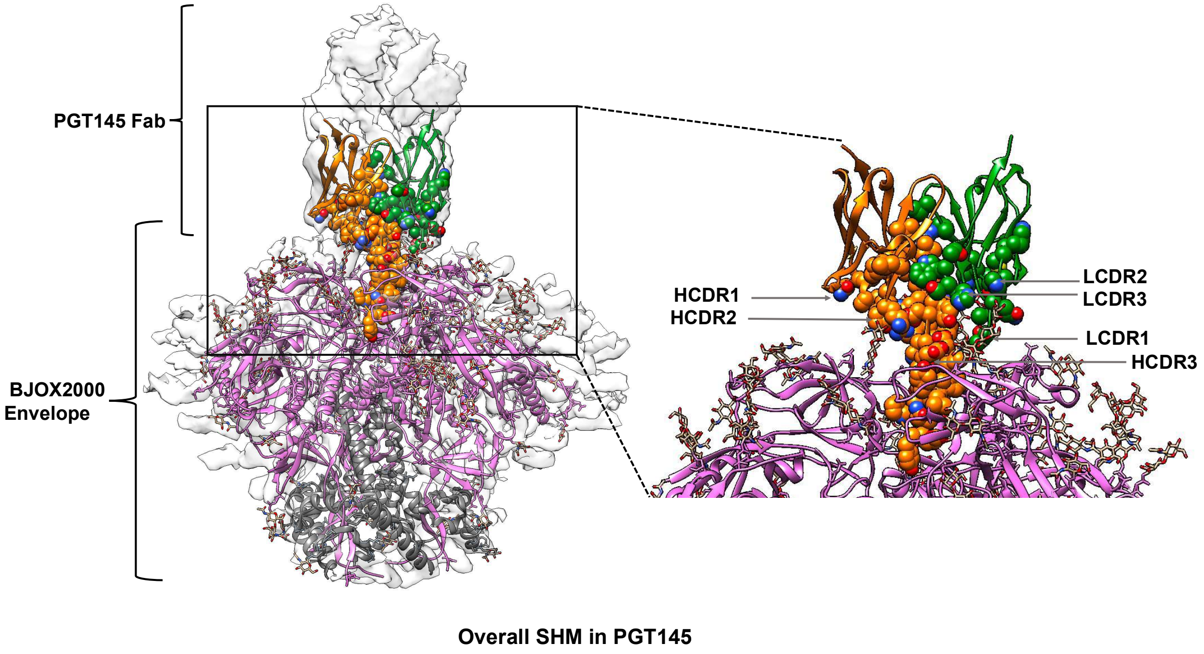

Figure S7. Sites of somatic hypermutations (SHM) in the PGT145 Fab shown with space filling atom models. The variable heavy and light chain of the PGT145 Fab are shown in BJOX2000+PGT145 complex structure. The variable light chain is colored green, and the variable heavy chain is colored orange. Regions that underwent somatic hypermutation (SHM) (shown as spheres) by comparison between the affinity matured PGT145 sequence and the inferred germline sequences (heavy chain: VH1-8*01, D4-17*01, JH6*02 and light chain: VK2D-28*01, JK1*01). SHM previously reported in Lee et al., Immunity, 2017)

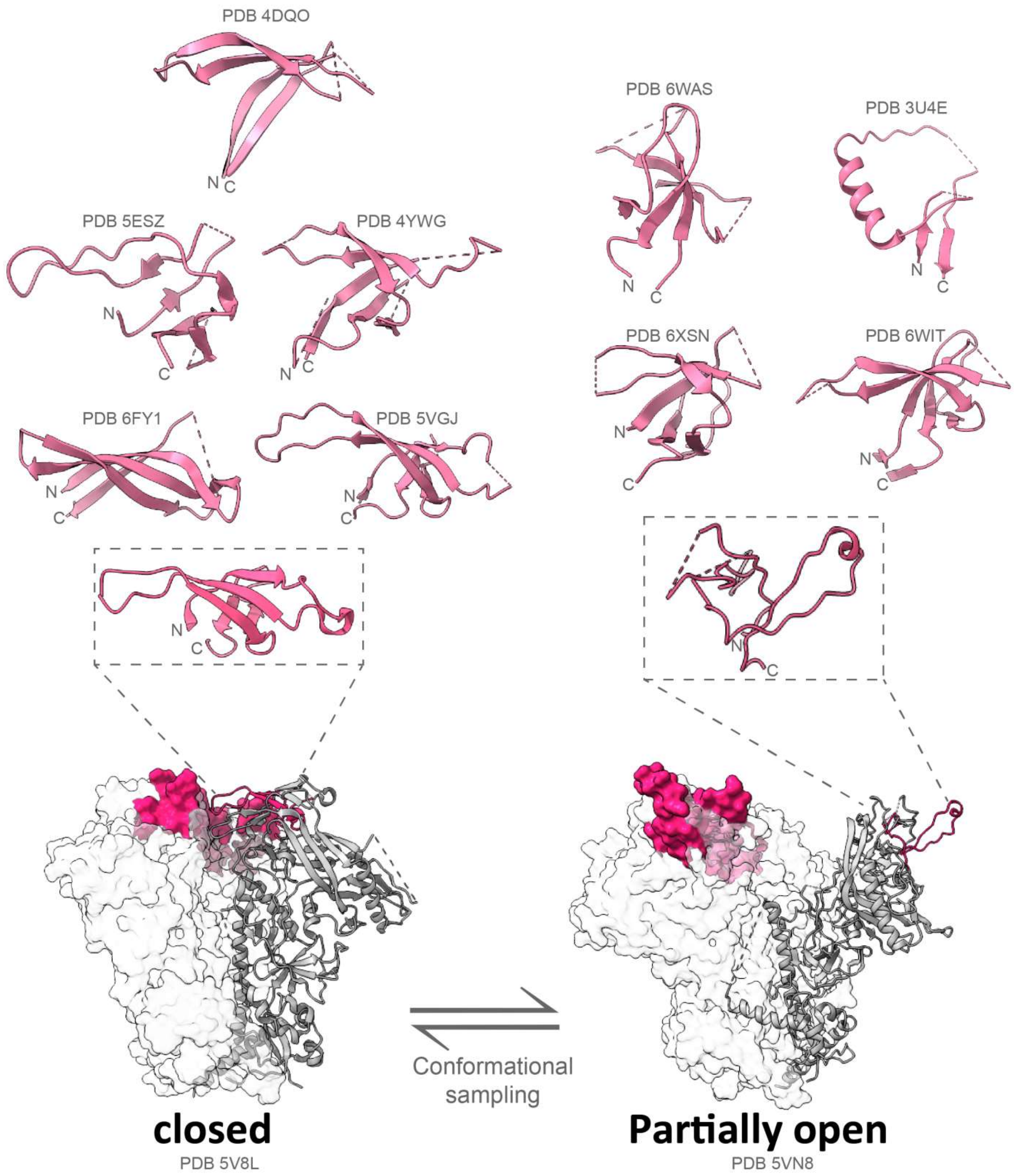

Figure S8. V1/V2 conformations on native-like Env trimers and presented on scaffold proteins. The V1/V2 apex is known to sample a wide range of conformations. On the bottom are Fab bound EM structures of SOSIP trimers in the closed conformation (PDB 5V8L) and a partially open conformation (PDB 5VN8) with 2 protomers displayed in white surface

representation, and 1 protomer displayed in gray ribbon representation with residues 126-196 spanning the V1/V2 region highlighted in red. Above the SOSIP trimers are V1/V2 crystal structures (residues 126-196) presented on scaffold protein and fAb bound to increase resolution and highlight the conformational plasticity of this region (PDB 4DQO, 5ESZ, 4YWG, 6FY1, 5VGJ, 6WAS, 3U4E, 6XSN, and 6WIT).

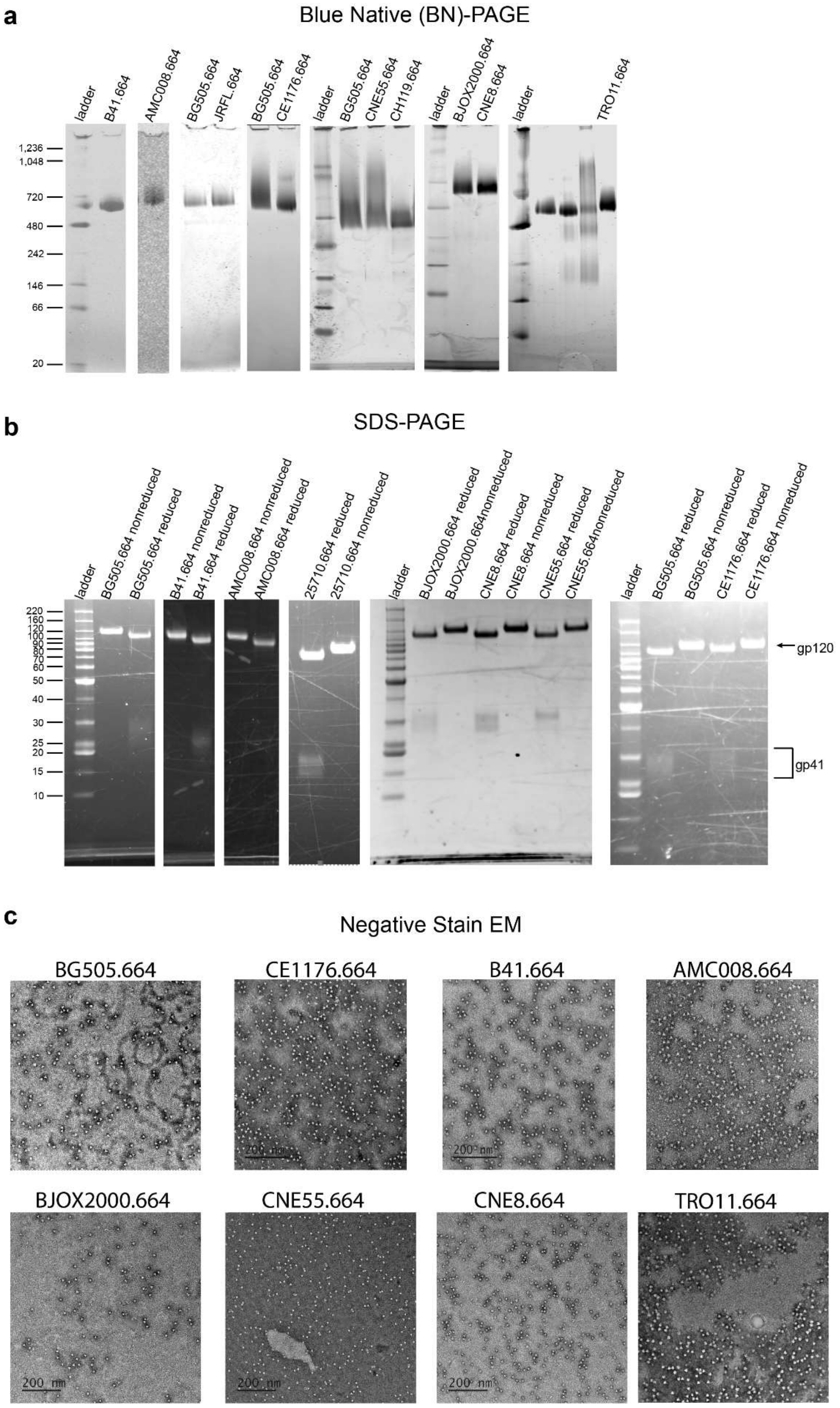
a
Blue Native (BN)-PAGE
ladder
B41.664
AMC008.664
BG505.664
JRFL.664
BG505.664
CE1176.664
ladder
BG505.664
CNE55.664
CH119.664
ladder
BJOX2000.664
CNE8.664
ladder
TRO11.664
1,236
1,048
720
480
242
146
66
20
b
SDS-PAGE
ladder
BG505.664 nonreduced
BG505.664 reduced
B41.664 nonreduced
B41.664 reduced
AMC008.664 nonreduced
AMC008.664 reduced
25710.664 reduced
25710.664 nonreduced
ladder
BJOX2000.664 reduced
BJOX2000.664nonreduced
CNE8.664 reduced
CNE8.664 nonreduced
CNE55.664 reduced
CNE55.664nonreduced
ladder
BG505.664 reduced
BG505.664 nonreduced
CE1176.664 reduced
CE1176.664 nonreduced
220
160
120
100
90
80
70
60
50
40
30
25
20
15
10
gp120
gp41
c
Negative Stain EM
BG505.664
CE1176.664
B41.664
AMC008.664
BJOX2000.664
CNE55.664
CNE8.664
TRO11.664
200 nm

Figure S9. SOSIP characterization by blue native (BN)-PAGE, SDS-PAGE, and Negative Stain EM (nsEM). Each SOSIP was characterized by BN-PAGE **(a)**, SDS-PAGE **(b)**, and negative stain EM **(c)** after purification and prior to HDX and BLI experiments. On the SDS-PAGE each sample was run non-reduced and reduced via addition of DTT. The single band in the non-reduced lane runs at a slightly higher MW due to the presence of an additional inter-subunit disulfide bond linking gp120 and gp41.

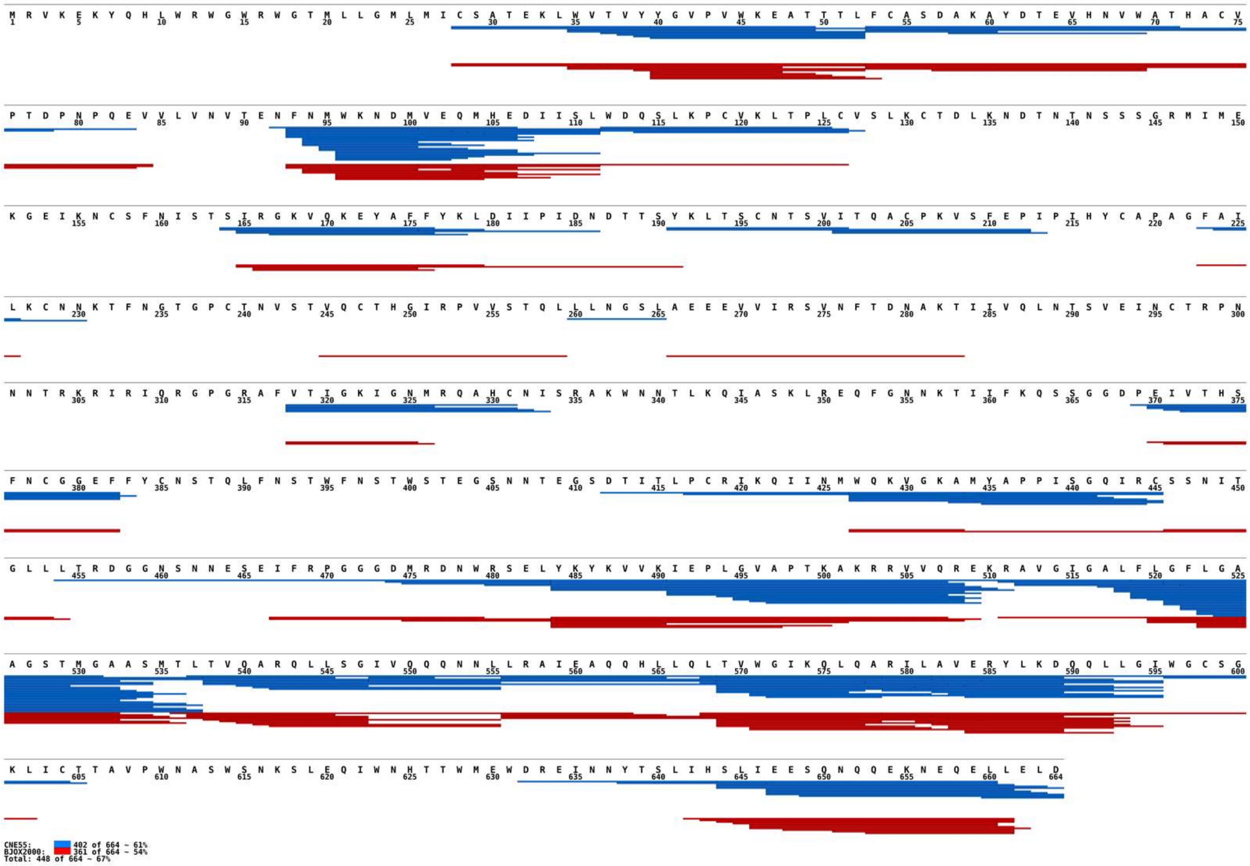

Figure S10. CNE55 and BJOX2000 SOSIP HDX-MS sequence coverage map. CNE55 (red) and BJOX2000 (blue) SOSIP trimer peptic peptides are shown as bars in the coverage map created using MSTools. HXB2 sequence and numbering are used. Missing coverage at the N-terminus is likely due to signal peptide cleavage of Env trimers post-translation.
